# Inhibiting centrosome clustering reduces cystogenesis and improves kidney function in autosomal dominant polycystic kidney disease

**DOI:** 10.1172/jci.insight.172047

**Published:** 2024-02-22

**Authors:** Tao Cheng, Aruljothi Mariappan, Ewa Langner, Kyuhwan Shim, Jay Gopalakrishnan, Moe R. Mahjoub

**Affiliations:** 1 Department of Medicine, Nephrology Division, Washington University School of Medicine, St. Louis, Missouri, USA.; 2 Institute of Human Genetics, Heinrich-Heine-Universität Düsseldorf, Düsseldorf, Germany.; 3 Institute of Human Genetics, Jena University Hospital, Friedrich Schiller University, Jena, Jena, Germany.; 4 Department of Cell Biology and Physiology, Washington University School of Medicine, St. Louis, Missouri, USA.

**Keywords:** Cell biology, Nephrology, Cytoskeleton

## Abstract

Autosomal dominant polycystic kidney disease (ADPKD) is a monogenic disorder accounting for approximately 5% of patients with renal failure, yet therapeutics for the treatment of ADPKD remain limited. ADPKD tissues display abnormalities in the biogenesis of the centrosome, a defect that can cause genome instability, aberrant ciliary signaling, and secretion of pro-inflammatory factors. Cystic cells form excess centrosomes via a process termed centrosome amplification (CA), which causes abnormal multipolar spindle configurations, mitotic catastrophe, and reduced cell viability. However, cells with CA can suppress multipolarity via “centrosome clustering,” a key mechanism by which cells circumvent apoptosis. Here, we demonstrate that inhibiting centrosome clustering can counteract the proliferation of renal cystic cells with high incidences of CA. Using ADPKD human cells and mouse models, we show that preventing centrosome clustering with 2 inhibitors, CCB02 and PJ34, blocks cyst initiation and growth in vitro and in vivo. Inhibiting centrosome clustering activates a p53-mediated surveillance mechanism leading to apoptosis, reduced cyst expansion, decreased interstitial fibrosis, and improved kidney function. Transcriptional analysis of kidneys from treated mice identified pro-inflammatory signaling pathways implicated in CA-mediated cystogenesis and fibrosis. Our results demonstrate that centrosome clustering is a cyst-selective target for the improvement of renal morphology and function in ADPKD.

## Abstract 2

Inhibition of centrosome clustering blocks cystic cell proliferation and improves renal morphology and function in autosomal dominant polycystic kidney disease cells and mice.

## Introduction

Autosomal dominant polycystic kidney disease (ADPKD) is the most common inherited monogenic kidney disorder, affecting approximately 13 million individuals worldwide. ADPKD is characterized by dysregulation of renal epithelial cell homeostasis and hyperproliferation of normally quiescent cells, which profoundly alters organ architecture and impairs renal function. It is well established that ADPKD is a ciliopathy, a disease caused by ciliary dysfunction. Cilia are microtubule-based organelles that play important chemo- and mechanosensory roles in cells (1). In the kidney, cilia are found on renal progenitor cells during development (2, 3) and quiescent epithelial cells lining the various segments of mature nephrons (4). The cilia protrude from the apical surface and are in contact with the extracellular environment, acting as a cellular sensor that regulates epithelial cell growth, homeostasis, and repair (5, 6). The assembly of cilia is templated by the centrosome, the main microtubule-organizing center in animal cells (7). Most cells in the human body, including in the kidney, contain a solitary centrosome and cilium, and cells have evolved tight regulatory mechanisms to ensure they contain only one of each organelle (8).

Recent studies have noted the presence of excess centrosomes (a phenomenon termed centrosome amplification; CA) in renal epithelial cells of patients with ADPKD (9, 10). Quantification of centrosome number in human ADPKD samples showed that approximately 14% of cystic cells contained excess centrosomes (9). Genetic ablation of *PKD1* in mice resulted in approximately 10% of cells containing extra centrosomes at P7 and P14 (9). Similarly, modulation of *PKD2* levels resulted in approximately 30% of cells with CA (10). Moreover, CA has been observed in livers of ADPKD mutant mice and patients with ADPKD (11). Roughly 15% of cystic cholangiocytes contain excess centrosomes in livers of the PCK rat and *Pkd2^WS25/–^* mouse model, compared with controls, as well as in patients with polycystic liver disease (11). Finally, loss of function in several other cyst-inducing genes has been shown to cause CA in kidney epithelial cells and even exacerbate the CA upon loss of *PKD1* (9, 12–16). Thus, several independent studies have demonstrated that CA occurs in cystic cells following loss of ADPKD-inducing genes.

CA is most commonly caused by centrosome overduplication, due to uncoupling of the centrosome biogenesis process from the cell cycle (8). Several studies have shown that loss of PKD1 function results in defective p21-dependent cell cycle regulation (17, 18). Thus, the most likely cause of CA following PKD1 loss of function is the defective coordination of cell division and the centrosome duplication cycles. CA is detrimental to cell physiology in multiple ways: (a) it causes abnormal mitotic spindle formation leading to chromosome segregation errors and genome instability (19, 20), (b) it disrupts cilia assembly and signaling (21), and (c) it results in increased secretion of cytokines and pro-inflammatory paracrine signaling factors (22, 23). Collectively, these changes enhance the proliferative capacity of cells (and tissues) associated with CA. This phenomenon has been extensively studied in the context of cancer, as CA has been shown to play a critical role in driving tumor cell proliferation and metastasis (23). Intriguingly, ADPKD is also associated with defects in genome stability, ciliary dysfunction, and changes in inflammatory signaling that may contribute to disease progression (24–34). Yet whether the CA observed in kidney samples of patients with ADPKD contributes to renal cystogenesis or how it affects kidney physiology have remained unclear.

To address these questions, we recently examined the consequences of CA during kidney development, homeostasis, and repair postinjury. Analysis of kidney specimens from patients with ADPKD showed that CA is highly prevalent in cystic epithelial cells but extremely rare in healthy kidneys (12), verifying prior observations. CA was evident early during cyst formation, and even present in normal-sized nephron tubule segments that had not become dilated or formed cysts, indicating this defect likely precedes cystogenesis. Using a genetic mouse model with which we can alter centrosome biogenesis in kidneys in vivo, we demonstrated that the formation of excess centrosomes indeed caused rapid-onset cystogenesis (12). Genetic induction of CA during embryonic kidney development in wild-type mice caused defects in the growth and differentiation of renal progenitors, resulting in kidneys that were smaller than normal at birth. Importantly, the kidneys developed cysts very rapidly, resulting in kidney failure and lethality by P15. Analysis of cells with CA showed defects in mitotic spindle morphology, ciliary assembly, and signaling pathways essential for growth and differentiation of renal progenitors, highlighting the mechanisms underlying the observed developmental phenotypes (12). Moreover, CA sensitized kidneys in adult mice, causing extensive cyst formation within 30 days after ischemic renal injury (12). Thus, the formation of excess centrosomes can be a driver of renal cyst formation during kidney development and homeostasis.

**Figure 1:**
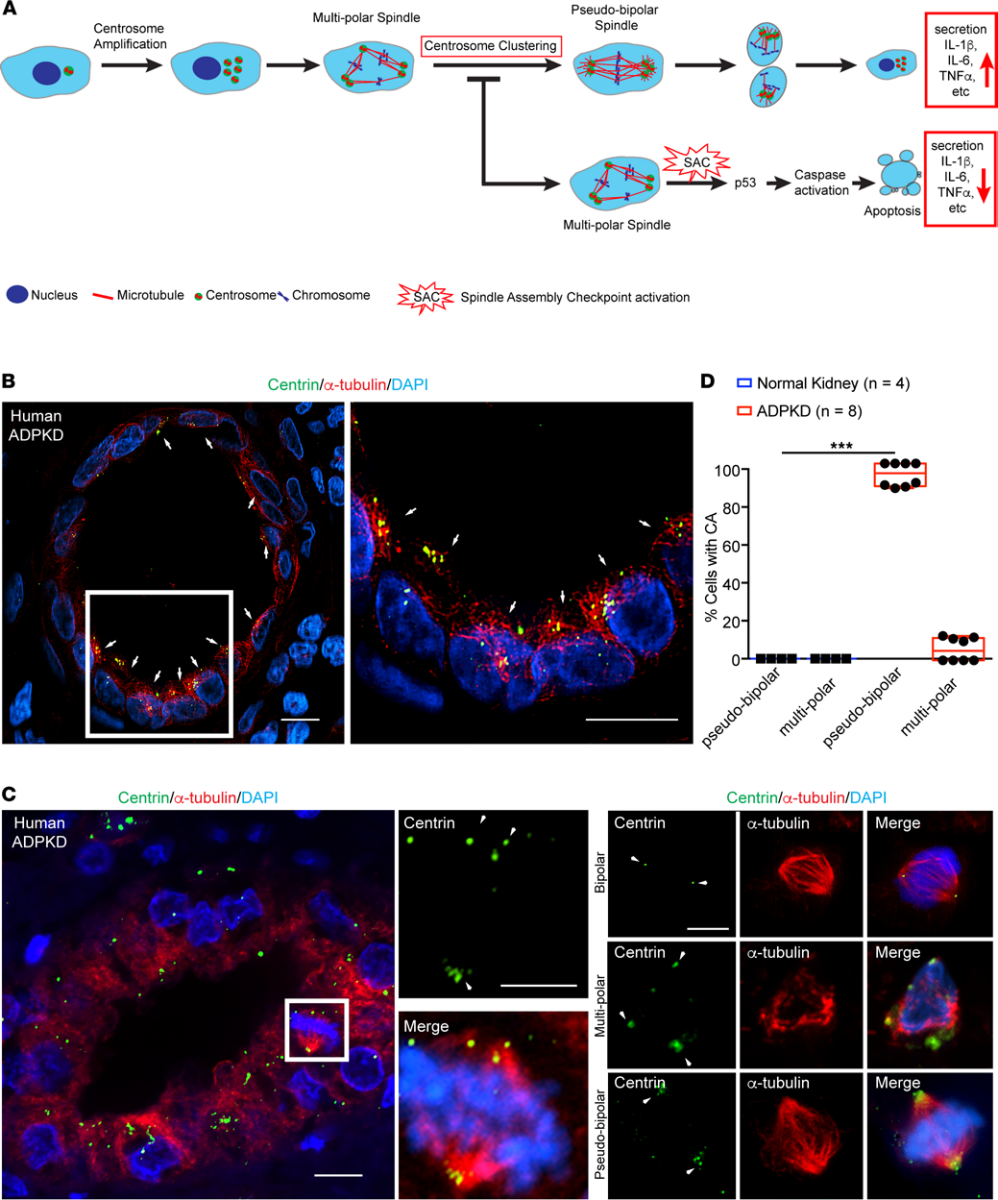
Human ADPKD cells with amplified centrosomes form pseudo-bipolar mitotic spindles. (**A**) Cartoon schematic of centrosome amplification (CA) and its consequences. Mitotic spindles in cells with excess centrosomes typically form more than 2 poles, leading to multipolar spindle configurations and activation of the spindle assembly checkpoint (SAC). This is followed by activation of the p53-mediated surveillance pathway, leading to caspase-mediated apoptosis. Centrosome clustering and the formation of pseudo-bipolar spindles comprise a survival mechanism adapted by some of these cells to avoid cell death. These cells subsequently demonstrate enhanced secretion of pro-inflammatory cytokines and growth factors. Inhibition of centrosome clustering can push cells toward the apoptotic pathway. (**B**) Immunofluorescence staining of human ADPKD kidney section with antibodies to highlight centrioles (centrin), microtubules (α-tubulin), and DNA (DAPI). Right panel shows magnified region indicated by white box. Arrows point to cyst-lining cells with amplified centrosomes. Scale bars = 10 μm. (**C**) Left panel: Representative immunofluorescence images of cells in mitosis in human ADPKD cyst. Tissue was stained with antibodies to highlight centrioles (centrin), spindle microtubules (α-tubulin), and DNA (DAPI). Right panel: Representative magnified immunofluorescence images of human ADPKD cystic cells in mitosis. Arrows point to centrosomes at spindle poles. Scale bars = 10 μm. Scale bar = 5 μm for the 2 magnified center images. (**D**) Quantification of spindle configurations in dividing cells from wild-type and ADPKD kidneys. Wild-type: *n* = 366 mitotic cells from 4 patient samples; ADPKD: *n* = 789 mitotic cells from 8 patient samples. ***P < 0.001 (1-way ANOVA).

In addition to the cell-intrinsic defects (i.e., genome instability and ciliogenesis) that confer a proliferative advantage to themselves, cells with amplified centrosomes have been found to secrete high levels of cytokines and growth factors, resulting in pro-inflammatory and proliferative paracrine signaling (22, 23). In essence, cells with CA can act as “signal amplifiers” that promote the growth of adjacent cells with normal centrosome number (22, 23). Thus, it has been proposed that eliminating cells with CA will attenuate both cell-intrinsic and -extrinsic effects. Recent studies in cancer cells support this theory. Amplified centrosomes typically cause the formation of multipolar mitosis and chromosome mis-segregation errors, leading cells to undergo mitotic catastrophe and cell death (Figure 1A). However, cancer cells with extra centrosomes achieve a pseudo-bipolar spindle configuration via a process termed “centrosome clustering,” a key adaptive mechanism by which they circumvent mitotic catastrophe and survive. Thus, inhibiting centrosome clustering to induce multipolar divisions, leading to negative selection of that population of cells, has been proposed as a strategy to counteract tumors with high incidences of CA (Figure 1A) (35). To achieve this, a number of drug discovery screens have identified selective small molecule inhibitors of centrosome clustering, some of which are currently in clinical trials for different types of human cancers (36, 37).

In this study, we sought to determine whether pharmacological inhibition of centrosome clustering in ADPKD mice can result in elimination of cells with CA, attenuation of progressive cystic growth, and improvement in kidney function in vivo.

## Results

### ADPKD cells with amplified centrosomes form pseudo-bipolar mitotic spindles.

We previously demonstrated that CA is prevalent in cyst-lining epithelia of ADPKD patient kidneys (12). Roughly 12% of cystic epithelial cells contain excess centrosomes (ranging from 5% to 23%, Figure 1B), and this phenotype is evident in approximately 43% of cysts (12). This range is consistent with what has been observed in numerous types of cancer cells with CA (38–42). To determine whether dividing cystic epithelia with CA clustered their centrosomes in mitosis, ADPKD patient kidney samples were immunostained with antibodies targeting centrosomes and microtubules. Mitotic cells with normal centrosome number formed bipolar spindles as expected, whereas cells with CA almost exclusively formed pseudo-bipolar spindle configurations in both metaphase and anaphase (Figure 1, C and D). This indicates that a robust centrosome clustering mechanism exists in ADPKD cystic epithelia, likely contributing to the ability of these cells to survive mitotic catastrophe, proliferate, and form cysts.

### Inducing CA accelerates the cystic disease phenotype in a slow-onset mouse model of ADPKD.

**Figure 2:**
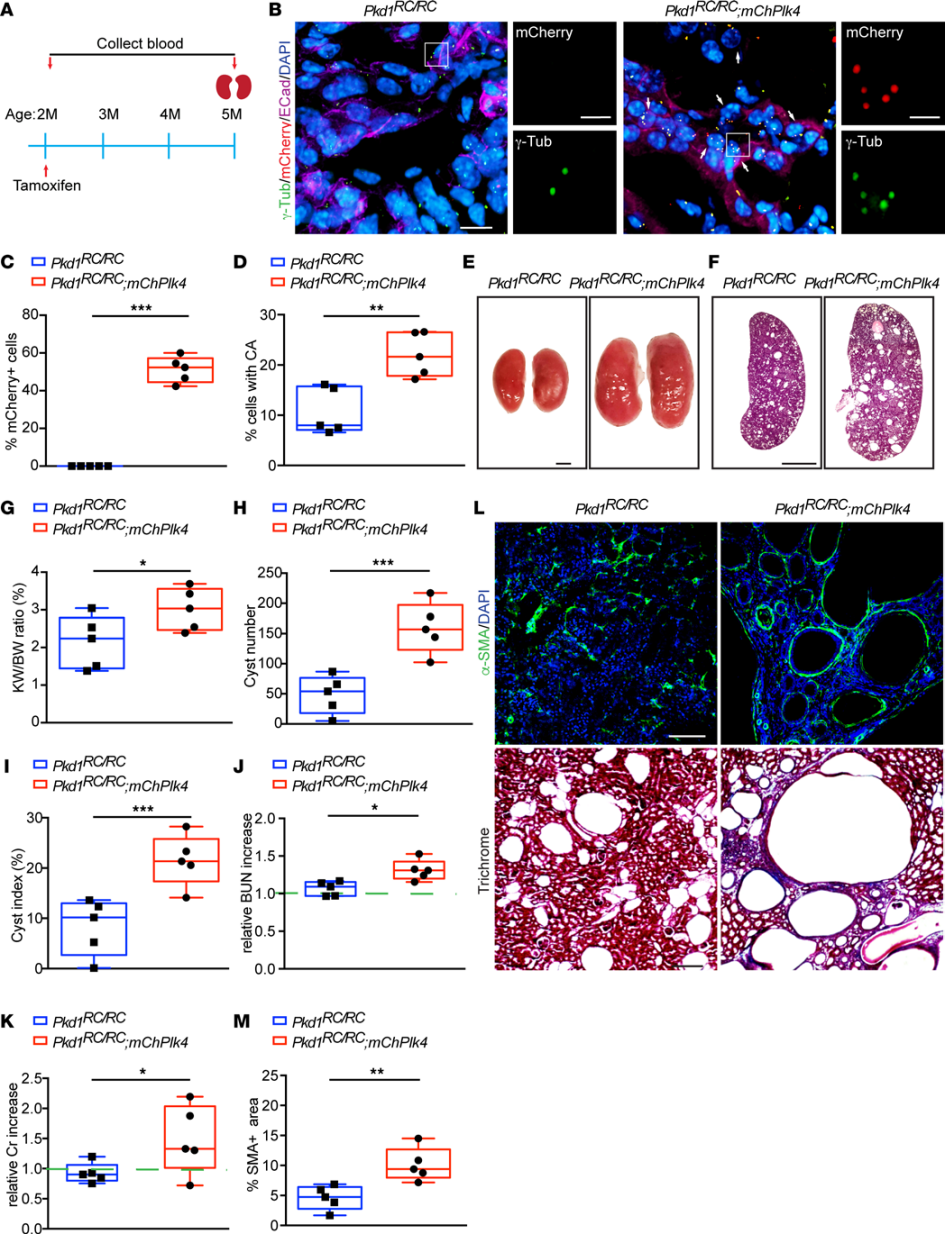
Induction of CA accelerates cystogenesis in slow-onset ADPKD mice. (**A**) Timeline of conditional induction of CA in *Pkd1^RC/RC^ mChPlk4* mice, including serum and kidney sample collection. (**B**) Immunofluorescence staining of kidney sections from 5-month-old *Pkd1^RC/RC^* and *Pkd1^RC/RC^ mChPlk4* mice using antibodies against mCherry, centrosomes (γ-tubulin), epithelial cells (E-cadherin), and DNA (DAPI). Arrows point to mCherry-positive cells with amplified centrosomes. Scale bars = 10 μm. Scale bar = 2 μm for all insets. (**C**) Quantification of the percentage of *mChPlk4*-positive cells and (**D**) the percentage of cyst-lining cells with excess (> 2) centrosomes. (**E** and **F**) Images of whole kidneys and H&E-stained sections of *Pkd1^RC/RC^* and *Pkd1^RC/RC^ mChPlk4* mice at 5 months of age. Scale bar = 2 mm (whole kidneys) and scale bar = 1 mm (H&E-stained sections). (**G**) Evaluation of kidney weight expressed as percentage of body weight at 5 months. (**H**) Quantification of cyst number and (**I**) fractional cyst area per kidney section. (**J**) Analysis of relative blood urea nitrogen (BUN) and (**K**) serum creatinine levels at 5 months. (**L**) Immunofluorescence staining (top) with α–smooth muscle actin (SMA; to mark myofibroblasts) and DNA (DAPI) and trichrome staining (bottom) of kidney sections from 5-month-old *Pkd1^RC/RC^* and *Pkd1^RC/RC^ mChPlk4* mice. Scale bar = 100 μm (SMA) and scale bar = 500 μm (trichrome). (**M**) Quantification of the fraction of α-SMA–positive area. *n* = 5 mice per group for all experiments. *P < 0.05, **P < 0.01, ***P < 0.001 (1-way ANOVA).

We previously demonstrated that inducing the formation of excess centrosomes in wild-type mice caused rapid cystogenesis shortly after birth (between P0 and P15) (12). Here, we sought to determine the synergistic effects of CA in conjunction with loss of function in ADPKD genes. We utilized a recently developed slow-onset ADPKD mouse model harboring a point mutation in *Pkd1* (43). The *Pkd1* variant p.Arg3277Cys (RC) in humans is hypomorphic and associated with typical slow-onset ADPKD in homozygosity (43). A mouse mimicking this variant (hereafter referred to as *Pkd1^RC/RC^*) is viable for at least 1 year with slowly progressive PKD. Cysts develop spontaneously, kidneys enlarge slowly up to 6 months of age, and cystogenesis accelerates beginning at 9 months. Interstitial renal fibrosis — a key factor that causes scarring and renal failure in ADPKD — and decline in filtration function start at 6 months of age and similarly accelerate at 9 months (43, 44). Quantification of centrosome number in 5-month-old *Pkd1^RC/RC^* mice showed that between 5% and 15% of cystic epithelial cells contained excess centrosomes (Figure 2D and Supplemental Figure 1C; supplemental material available online with this article; https://doi.org/10.1172/jci.insight.172047DS1), consistent with what we observed in human ADPKD samples. Overall, this mouse model effectively mimics the pathophysiological features of slow-onset progressive cystogenesis of human ADPKD, provides a large window of time to study the disease progression, and has been used extensively for preclinical testing of potential therapeutic compounds (26, 43, 45–58).

To cause elevated levels of CA in the ADPKD mutant background, *Pkd1^RC/RC^* were crossed with a transgenic model that allows for conditional expression of mCherry-tagged Plk4 (*mChPlk4*), which we previously used to drive CA in wild-type mouse kidneys (12). Expression of Plk4 was induced in *Pkd1^RC/RC^ mChPlk4* animals beginning at 2 months of age (Figure 2A) when cystogenesis is mild, the kidney weight–to–body weight (KW/BW) ratio is normal, and kidney function is mostly unaffected (43). This provided a means to examine the effects of enhanced CA at stages when the ADPKD phenotype is mild in this model. Immunofluorescence staining of kidneys isolated after 3 months verified that approximately 55% of cells expressed mCherry-Plk4 (Figure 2, B and C). Quantification of cells with CA showed a 2.8-fold increase in *Pkd1^RC/RC^ mChPlk4* animals compared with *Pkd1^RC/RC^* mice alone (Figure 2D). The kidneys became larger, with an approximately 35% increase in KW/BW (Figure 2, E and G, and Supplemental Figure 1A). Quantification of cyst number and fractional cyst area showed a near 175% and 120% increase, respectively (Figure 2, F, H, I, and Supplemental Figure 1B). Analysis of blood urea nitrogen (BUN) and serum creatinine indicated elevated levels compared with the baseline in *Pkd1^RC/RC^* mice, highlighting a decline in kidney filtration function upon CA (Figure 2, J and K, and Supplemental Figure 1, D and E). This was accompanied by an approximately 102% increase in interstitial fibrosis (Figure 2, L and M). Together, these data indicate that CA can significantly accelerate the cystic disease phenotype in slow-onset ADPKD mice.

### Inhibition of centrosome clustering in ADPKD cells activates the spindle assembly checkpoint and blocks proliferation.

Next, we sought to test whether blocking centrosome clustering in ADPKD cells with excess centrosomes can halt their proliferation, as has been proposed for cancer cells. Three inhibitors of centrosome clustering that have recently been identified in high-throughput in vitro screens — and tested in mice — are CCB02, AZ82, and PJ34. CCB02 is a novel tubulin-binding molecule that competes for the tubulin-binding site of centrosomal P4.1-associated protein (CPAP), forcing the extra centrosomes in a cell to nucleate high levels of microtubules prior to mitosis and thus prevent them from clustering (59). Treatment of various cancer cells with CCB02 reduced the fraction with supernumerary centrosomes in vitro and resulted in decreased cell proliferation. Notably, CCB02 had no detrimental effect on dividing cells with the normal complement of centrosomes (59). Oral administration of CCB02 to mice bearing human cancer xenografts resulted in reduction of centrosome-amplified tumor cells in vivo. Blocking centrosome clustering caused significant inhibition of tumor growth at doses well tolerated by the animals (59). AZ82 is an inhibitor of KIFC1, a kinesin-14 family motor protein that has been shown to play a critical role in centrosome clustering in various types of cancer cells (60, 61). PJ34 is a phenanthrene-derived PARP1 inhibitor that can suppress the expression and activity of the kinesin KIFC1 (62–64). PJ34 treatment blocks centrosome clustering, induces multipolar spindle formation in cells with CA (62), and results in decreased breast cancer cell proliferation. Importantly, the specificity of these inhibitors toward their respective targets has already been established by screening against large libraries of proteins and shown not to impact the proliferation of cells with normal centrosome number in vitro and in vivo (59–62). Collectively, these findings indicate that blocking centrosome clustering with these inhibitors is a viable approach to reduce the proliferation of cells with CA in vivo.

**Figure 3:**
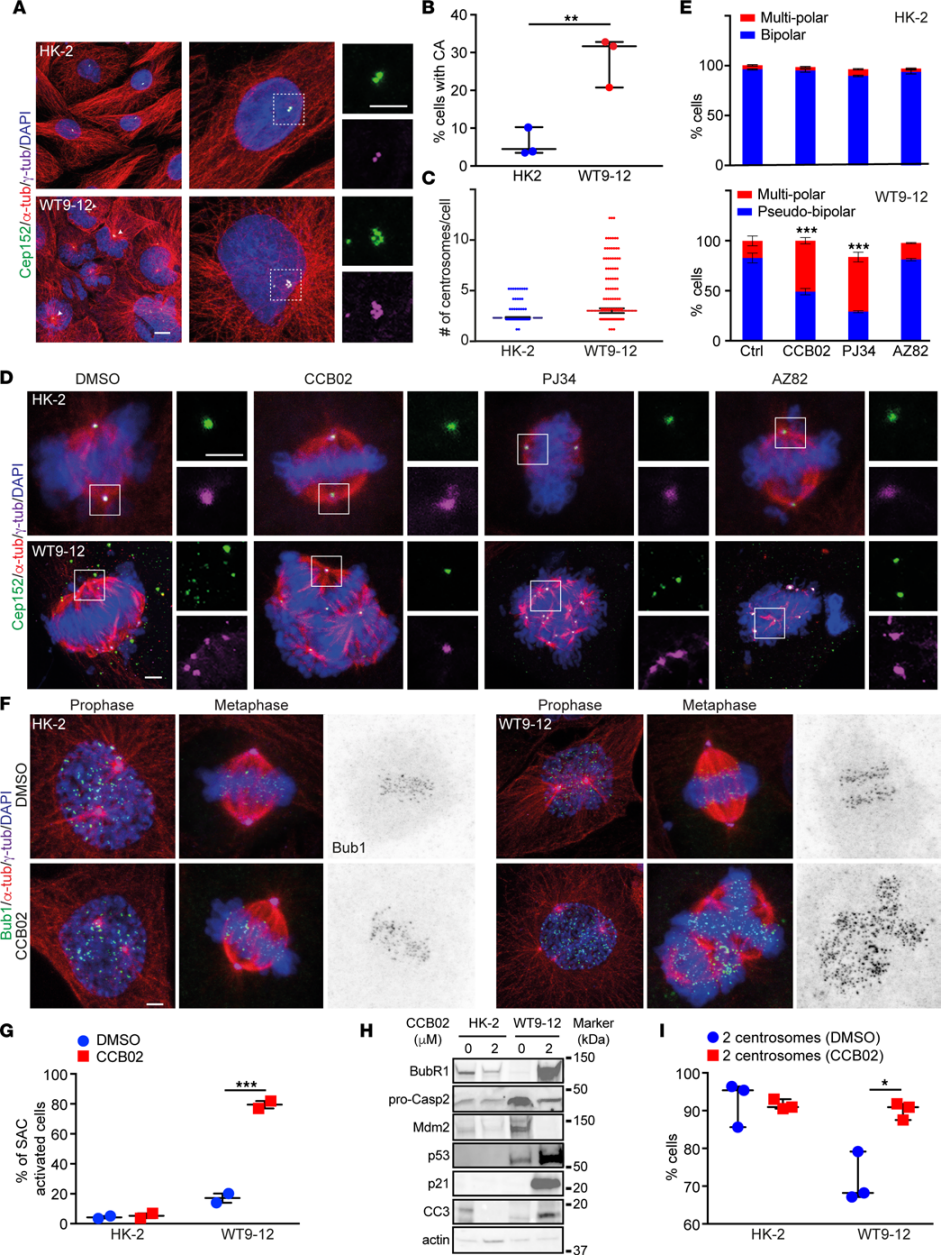
Inhibition of centrosome clustering in ADPKD cells promotes multipolar spindle formation and activates the SAC. (**A**) Immunofluorescence staining of interphase wild-type (HK-2) and PKD1-null (WT9-12) cells for centrioles (Cep152), centrosomes (γ-tubulin), microtubules (α-tubulin), and DNA (DAPI). Scale bar = 2 μm. Scale bar = 2 μm for all insets. (**B**) Percentage of cells with amplified centrosomes. *n* = 293 (HK-2) and 374 (WT9-12) cells. (**C**) Distribution of the number of excess centrosomes per cell. *n* = 301 (HK-2) and 322 (WT9-12) cells. (**D**) Immunofluorescence staining of mitotic wild-type (HK-2) and *PKD1*-null (WT9-12) cells for centrioles (Cep152), centrosomes (γ-tubulin), microtubules (α-tubulin), and DNA (DAPI). Scale bar = 2 μm. Scale bar = 2 μm for all insets. (**E**) Top graph shows the percentage of wild-type cells that form bipolar (cells containing normal centrosome number) or multipolar (cells with > 2 centrosomes) spindles. Bottom graph shows the percentage of *PKD1*-null cells with CA that formed pseudo-bipolar (clustered centrosomes) versus multipolar (declustered centrosomes). For HK-2 cells: *n* = 207 (control), 674 (CCB02), 174 (PJ34), 101 (AZ82); for WT9-12 cells: *n* = 393 (control), 449 (CCB02), 205 (PJ34), 189 (AZ82). (**F**) Immunofluorescence staining of mitotic wild-type and PKD1-null cells for centrosomes (γ-tubulin), microtubules (α-tubulin), Bub1, and DNA (DAPI). Grayscale images provide improved contrast of the Bub1 staining. Scale bar = 2 μm. (**G**) Percentage of cells showing Bub1 accumulation in mitosis. For HK-2 cells: *n* = 167 (DMSO), 133 (CCB02); for WT9-12 cells: *n* = 180 (DMSO), 278 (CCB02). Results are from 2 experiments. (**H**) Immunoblot of wild-type and *PKD1*-null cells treated with vehicle or CCB02. (**I**) Percentage of wild-type and PKD1-null cells containing normal centrosome number following treatment with CCB02. *n* = 371 (HK-2) and 264 (WT9-12) cells. For all experiments, cells were incubated with each inhibitor for 24 hours. Results are from 3 experiments. *P < 0.05, **P < 0.01, ***P < 0.001 (2-way ANOVA).

Using these 3 compounds, we first sought to test whether inhibition of centrosome clustering can induce the formation of multipolar mitotic spindles and mitotic arrest in ADPKD cells in vitro. Quantification of centrosome number in wild-type human kidney cells (HK-2) and *PKD1*-null cells (WT9-12) (65, 66) showed that roughly 28% of ADPKD cells contained excess centrosomes, significantly higher than the control cells (Figure 3, A–C). To eliminate the possibility that the observed CA was due to accumulation of secondary mutations in the immortalized WT9-12 cells, we also obtained 3 independent primary ADPKD cystic cell lines (isolated from 3 separate patients). Quantification of centrosome number indicated that the percentage of cells with CA ranged between approximately 20% and 40% (Supplemental Figure 2, A and B), consistent with the range observed in WT9-12 (Figure 3, A–C), as well as ADPKD human kidneys, ADPKD mouse models, and isolated cystic cells from patients with ADPKD (9, 12–15). As *PKD1*-null cells with CA entered mitosis, the vast majority (>80%) clustered their centrosomes and displayed a pseudo-bipolar spindle configuration (Figure 3, D and E).

To assess how mitotic cells with excess centrosomes respond to the centrosome clustering inhibitors, both control and *PKD1*-null cells were treated with CCB02, PJ34, or AZ82 for 24 hours (Figure 3D). Consistent with previous reports showing no adverse effects on spindle formation in cells with normal centrosome number (59–61, 63, 64), both control and *PKD1*-null cells with 2 centrosomes predominantly formed bipolar spindles in metaphase when treated with the inhibitors (Figure 3, D and E). In contrast, *PKD1*-null cells with CA formed multipolar and disorganized spindles in mitosis (Figure 3, D and E). This was evident upon treatment with CCB02 and PJ34 but not significantly with AZ82. Since the AZ82 inhibitor showed cellular toxicity and caused lethality when tested in ADPKD mice (data not shown), we focused solely on CCB02 and PJ34 for the remainder of this study. In CCB02- and PJ34-treated cells, centrosomes were present at the majority of excess spindle poles, and there was a concurrent decrease in the proportion of metaphase cells with bipolar spindles (Figure 3E).

Next, we tested whether CCB02-induced centrosome declustering caused a mitotic delay, activation of the SAC, and elimination of cells with CA from the population. Control and *PKD1*-null cells were incubated for 24 hours with either vehicle or CCB02 and cells stained using antibodies against budding uninhibited by benzimidazoles 1 (Bub1), which accumulates on unattached kinetochores and acts as a surrogate marker of SAC activation (59, 67). First, we verified that cells with either normal or excess centrosomes showed an accumulation of Bub1 in prophase (Figure 3F), which is expected because of the unattached kinetochores at this stage of the cell cycle (68). Cells with bipolar and pseudo-bipolar spindles showed a decrease in these levels in metaphase (Figure 3, F and G). In contrast, CCB02-treated *PKD1*-null cells with multipolar spindles displayed significant accumulation of Bub1 in metaphase (Figure 3, F and G), indicating that inhibition of centrosome clustering activates the SAC specifically in extra centrosome–containing cells.

To further verify that inhibition of centrosome clustering causes activation of the SAC, we also performed immunoblotting of lysates prepared from cells treated with CCB02. Indeed, inhibition of clustering caused an increase in the expression of BubR1, another key marker that is elevated upon SAC activation (Figure 3H) (69). Subsequently, supernumerary centrosomes then cause activation of the PIDDosome, which promotes the autocatalytic, proximity-induced activation of caspase-2 (70). Probing for pro-caspase-2 showed a decrease in levels upon CCB02 treatment (Figure 3H), as predicted. Active caspase-2 cleaves mouse double minute 2 (MDM2), a negative regulator of p53 stability, and thereby stabilizes p53 (70, 71). Consistent with this, we found that inhibition of centrosome clustering resulted in loss of MDM2 protein and stabilization of p53. Activated p53 following CA promotes either cell death (via activation of the caspase-3 pathway) and/or cell cycle arrest (via modulation of p21 function) (8, 70, 72). Immunoblotting for p21 and cleaved caspase-3 showed elevated levels of both proteins, signifying activation of both pathways. Importantly, all of these changes were observed specifically in the ADPKD cells, not the control cells treated with CCB02 (Figure 3H). Finally, incubation with centrosome clustering inhibitors showed an increase in the fraction of cells with the normal complement of 2 centrosomes (Figure 3I), indicating that the cells with CA (Figure 3B) had been eliminated from the population. Collectively, these data indicate that inhibiting centrosome clustering in ADPKD cells promotes formation of multipolar mitotic spindles, activation of the SAC, and reduced abundance of cells with CA due to cell cycle arrest and apoptosis.

### Inhibition of centrosome clustering attenuates cystic disease progression in vivo.

**Figure 4:**
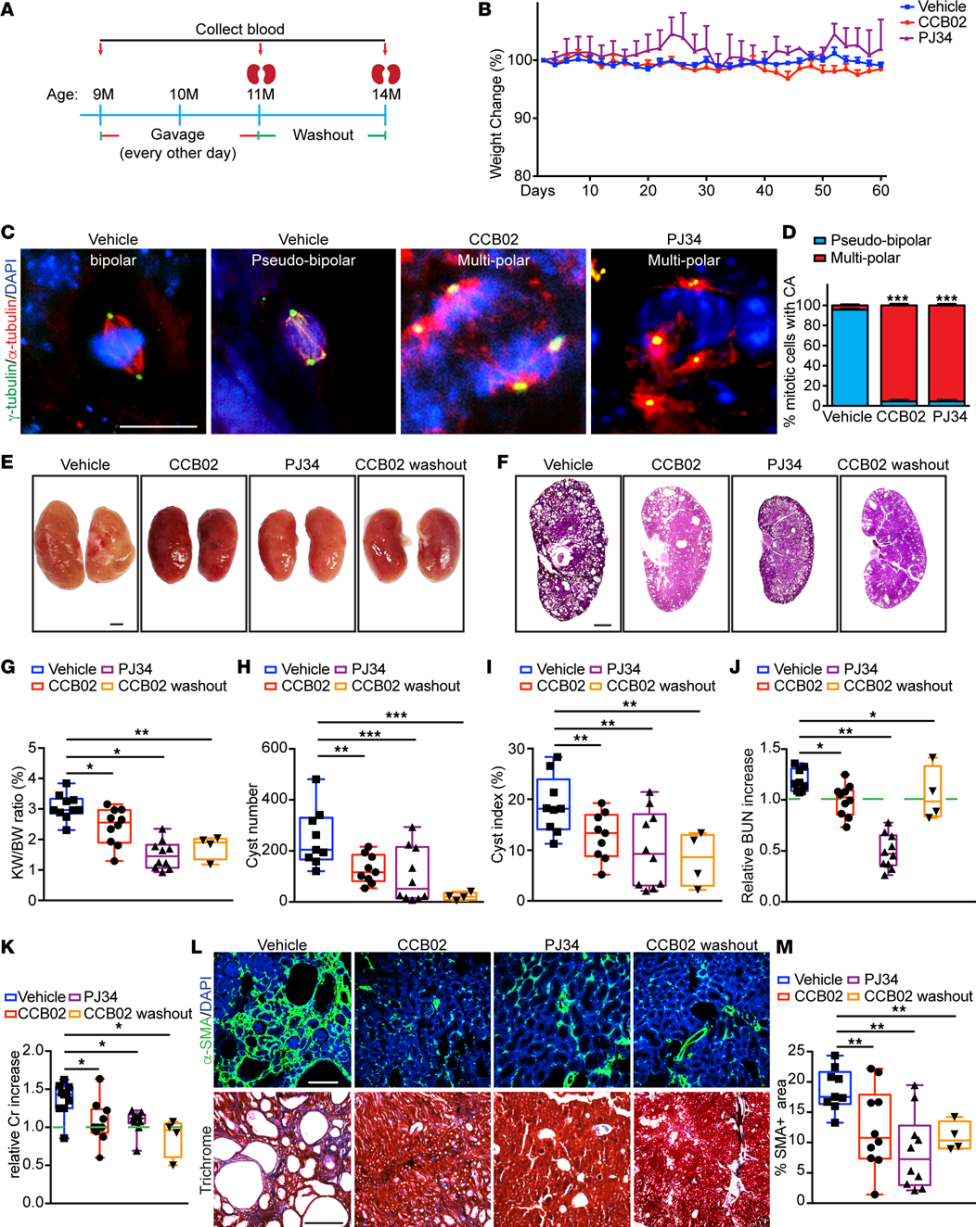
Inhibition of centrosome clustering attenuates disease progression during rapid stages of cytogenesis in vivo. (**A**) Schematic representation of CCB02 and PJ34 treatment scheme and washout in *Pkd1^RC/RC^* mice. (**B**) Quantification of relative weight change during the treatment timeline. (**C**) Immunofluorescence staining of kidney sections from *Pkd1^RC/RC^* mice at 11 months with antibodies to highlight centrosomes (γ-tubulin), spindle microtubules (α-tubulin), and DNA (DAPI). Scale bar = 10 μm. (**D**) Quantification of the percentage of spindle configurations at metaphase. *n* = 201 cells (vehicle group), 134 (CCB02 group), and 117 (PJ34 group). (**E** and **F**) Images of whole kidneys and H&E-stained sections of *Pkd1^RC/RC^* mice at 11 months of age after treatment with CCB02 or PJ34 and postwashout (14 months). Scale bar = 2 mm (whole kidneys) and scale bar = 1 mm (H&E-stained sections). (**G**) Evaluation of kidney weight expressed as percentage of body weight. (**H**) Quantification of cyst number and (**I**) fractional cyst area per kidney section in treated and untreated mice. (**J**) Analysis of relative blood urea nitrogen (BUN) and (**K**) serum creatinine levels at 11 months and postwashout (14 months). (**L**) Immunofluorescence staining (top) with α–smooth muscle actin (SMA; myofibroblasts) and DNA (DAPI) and trichrome staining (bottom) of kidney sections following the indicated treatment regimen. Scale bar = 100 μm (SMA) and scale bar = 500 μm (trichrome). (**M**) Quantification of the fraction of α-SMA–positive area. *n* = 10 mice each for vehicle, CCB02, and PJ34; *n* = 4 animals for CCB02 washout group. *P < 0.05, **P < 0.01, ***P < 0.001 (1-way ANOVA).

To determine the effects of centrosome clustering inhibitors on cyst growth in vivo, *Pkd1^RC/RC^* mice were first treated with CCB02 or PJ34 starting at 9 months of age, the stage when expansion of cystic area, cyst number, and KW/BW begin to accelerate (43). Mice were administered CCB02 or PJ34 every 2 days for a total period of 2 months, which was well tolerated by the animals (Figure 4, A and B). Immunofluorescence analysis of kidney sections from control (vehicle only) *Pkd1^RC/RC^* mice indicated that the majority (~95%) of cells with CA displayed a clustered configuration (Figure 4, C and D). In contrast, both CCB02 and PJ34-treated kidney cells with CA predominantly contained multipolar spindles (Figure 4, C and D), indicating that the centrosome clustering inhibitors are also active in vivo. Remarkably, treatment with either inhibitor resulted in a significant decrease in kidney size, cyst index, and cyst number compared with control (Figure 4, E–I, and Supplemental Figures 3 and 4). Quantification of BUN and serum creatinine showed elevated levels relative to the baseline in control *Pkd1^RC/RC^* mice over that 2-month period, whereas mice treated with either inhibitor maintained significantly lower levels of both (Figure 4, J and K, and Supplemental Figure 5, A and B). In addition, there was a concurrent decrease in interstitial fibrosis (Figure 4, L and M). Thus, treatment with centrosome clustering inhibitors during the rapid cystogenesis stage in this ADPKD mouse model caused improvement in kidney morphology and function.

Another approach to test the effects of the clustering inhibitors during rapid cyst formation is to utilize the *Pkd1^RC/RC^ mChPlk4* mice, which develop cysts rapidly upon induction of CA (Figure 2). CA was induced in 2-month-old *Pkd1^RC/RC^ mChPlk4* mice, and then animals were treated with CCB02 or vehicle for 6 weeks (Supplemental Figure 6A). Similar to the *Pkd1^RC/RC^* mice, treatment with CCB02 resulted in reduced cyst index and cyst number compared with control (Supplemental Figure 6, B–E). Quantification of BUN, serum creatinine, and interstitial fibrosis similarly showed improved preservation of kidney function over the 6-week period (Supplemental Figure 6, F–K). These data verify that inhibiting centrosome clustering during the rapid cyst expansion stage results in reduced cyst growth, improved kidney morphology, and preserved renal function.

**Figure 5:**
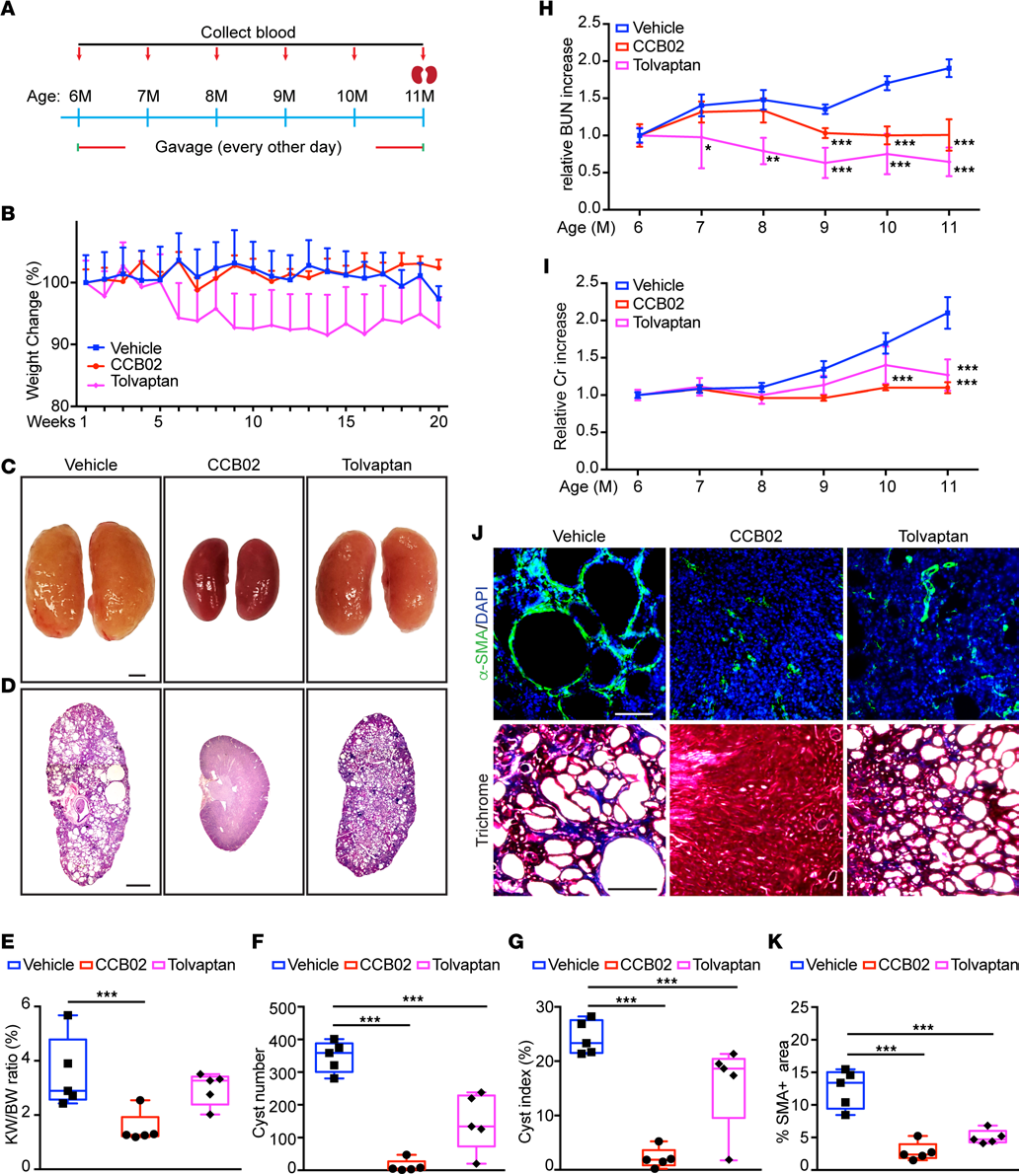
Inhibition of centrosome clustering at earlier stages of cystogenesis shows stronger effects on disease progression. (**A**) Schematic representation of CCB02 and tolvaptan treatment scheme in *Pkd1^RC/RC^* mice. (**B**) Quantification of relative weight change during the treatment timeline. (**C** and **D**) Images of whole kidneys and H&E-stained sections of *Pkd1^RC/RC^* mice at 11 months of age after treatment with CCB02 or tolvaptan. Scale bar = 2 mm (whole kidneys) and scale bar = 1 mm (H&E-stained sections). (**E**) Evaluation of kidney weight expressed as percentage of body weight. (**F** and **G**) Quantification of cyst number and fractional cyst area per kidney section in treated and untreated mice. (**H**) Analysis of relative changes in blood urea nitrogen (BUN) and (**I**) serum creatinine levels. (**J**) Immunofluorescence staining (top) with α-SMA (myofibroblasts) and DNA (DAPI) and trichrome staining (bottom) of kidney sections following the indicated treatment regimen. Scale bar = 100 μm (SMA) and scale bar = 500 μm (trichrome). (**K**) Quantification of the fraction of α-SMA–positive area. *n* = 5 mice per group for all experiments. *P < 0.05, **P < 0.01, ***P < 0.001 (1-way ANOVA).

Next, we sought to determine whether treatment with centrosome clustering inhibitors beginning earlier, and for a longer period, would yield better outcomes with regard to cystogenesis and kidney function. *Pkd1^RC/RC^* mice at 6 months of age displayed relatively mild ADPKD phenotypes, with minimal increase in kidney volume, and cyst size and number, and no significant decline in renal function (43, 44, 53). Using a similar dosing scheme, mice were administered either vehicle or CCB02 beginning at 6 months of age every 2 days for a total of 5 months (Figure 5A). In parallel, *Pkd1^RC/RC^* mice were given tolvaptan, a selective vasopressin receptor 2 antagonist that is currently the only FDA-approved drug for treatment of ADPKD (73, 74) and has a different mechanism of action than centrosome clustering inhibitors. Similar to the 2-month treatment plan, the mice showed no adverse effects to CCB02 whereas tolvaptan-treated animals showed substantial weight loss over that 5-month period (Figure 5B), which has been previously reported as a significant side effect of this compound (74, 75). Treatment with CCB02 resulted in preservation of kidney size, and reduced cyst index and cyst number, compared with control *Pkd1^RC/RC^*, better than the tolvaptan-treated group (Figure 5, C–G, and Supplemental Figure 7, A and B). Importantly, these differences were more pronounced when compared with the 2-month treatment plan (Figure 4, E–I, and Supplemental Figures 3 and 4). Quantification of BUN, serum creatinine, and interstitial fibrosis similarly showed improved preservation of kidney function over the 5-month period (Figure 5, H–K, and Supplemental Figure 7, C and D) when compared with the 2-month treatment plan (Figure 4, J–M, and Supplemental Figure 5, A and B). The maintenance of kidney function upon CCB02 treatment was similar to the effect of tolvaptan, highlighting the potential for centrosome clustering inhibitors in reducing disease development and severity over prolonged periods.

Finally, we sought to determine whether short-term treatment of ADPKD mice with centrosome clustering inhibitors, followed by a period of no drug administration, would provide beneficial long-term effects. Nine-month-old *Pkd1^RC/RC^* mice were given CCB02 every other day for 60 days, the treatment was stopped, and the mice were followed for 3 months until they reached 14 months of age (Figure 4A). Analysis of kidney morphology, function, and fibrosis showed that the dramatic improvements observed at 11 months were maintained, even when the drug was no longer administered (Figure 4, E–M). In sum, our results indicate that inhibiting centrosome clustering in slow-onset ADPKD mice results in reduced cyst growth, improved kidney morphology, and preserved renal function, which are evident even after drug treatment is halted.

### Centrosome clustering inhibitors promote apoptosis of cells with CA and reduce inflammatory signaling.

**Figure 6:**
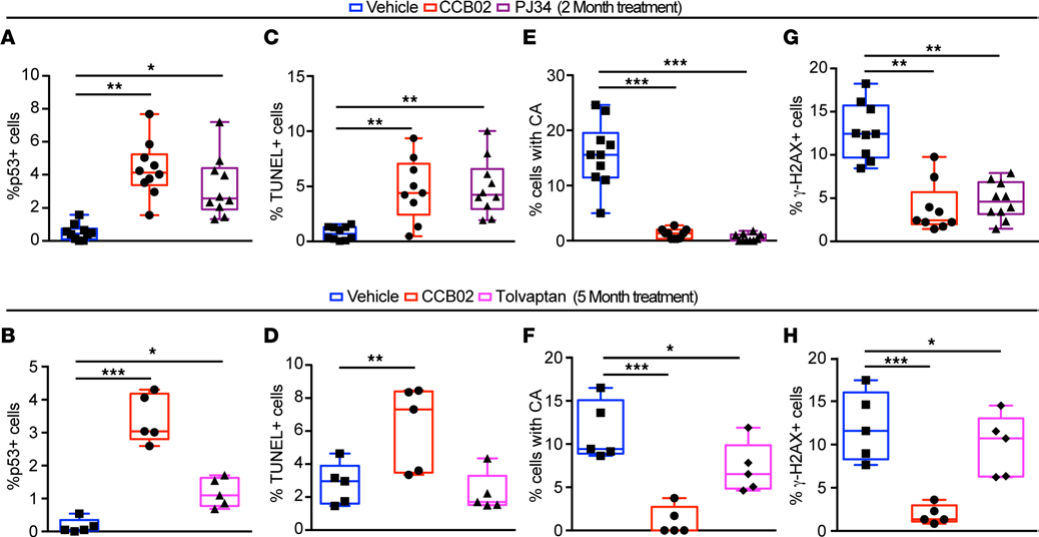
Centrosome clustering inhibitors promote p53-mediated apoptosis and attenuate pro-inflammatory signaling pathways. (**A** and **B**) Quantification of p53-positive nuclear staining in kidneys of *Pkd1^RC/RC^* mice treated with CCB02 or PJ34 for 2 months or with CCB02 or tolvaptan for 5 months. (**C** and **D**) Quantification of TUNEL staining in *Pkd1^RC/RC^* kidney sections treated as indicated. (**E** and **F**) Quantification of the percentage of cyst-lining cells with excess (> 2) centrosomes. (**G** and **H**) Quantification of γ-H2AX–positive cells after treatment for 2 or 5 months. *n* = 10 mice per group (2-month treatment); *n* = 5 mice per group (5-month treatment).

Spindle formation defects caused by CA often result in activation of mitotic stress and DNA damage sensors, p53-mediated cell cycle arrest, and ultimately cell death via induction of apoptotic signaling pathways (12, 59, 76–79). To determine whether inhibition of centrosome clustering promotes p53-mediated cell death, kidneys of *Pkd1^RC/RC^* mice were analyzed. We noted a significant increase in total p53 (Supplemental Figure 9A) and nuclear accumulation of p53 in mice treated with CCB02 or PJ34 for both 2 and 5 months, compared with control untreated animals (Figure 6, A and B, and Supplemental Figure 8, A and B). This corresponded with an increase in cell death as noted by TUNEL staining (Figure 6, C and D, and Supplemental Figure 8, C and D) and a concomitant reduction in the number of cells with amplified centrosomes (Figure 6, E and F, and Supplemental Figure 8, E and F). As cells with CA divide, the resulting daughter cells often display low levels of chromosome mis-segregation and aneuploidy, which activates the DNA damage response pathway (80). Indeed, untreated *Pkd1^RC/RC^* mice displayed elevated levels of γ-H2AX (Figure 6, G and H, and Supplemental Figure 8, G and H), a surrogate marker of the DNA damage response. In contrast, there was a significant reduction in γ-H2AX staining following treatment with CCB02 or PJ34. Finally, we quantified the proliferation index of the cyst-lining epithelia upon treatment with CCB02 and PJ34. Quantification of the percentage of PCNA^+^ cyst-lining cells suggested a significant reduction in kidneys treated with CCB02 and PJ34 compared with control (Supplemental Figure 9, B and C). We interpret these data to suggest that inhibiting centrosome clustering causes elimination of cells with CA from the population via reduced proliferation and increased apoptosis.

Finally, we tested whether loss of cells with amplified centrosomes would attenuate their paracrine mediated effects. Recent studies have shown that CA promotes the secretion of factors with proliferative, pro-inflammatory, and pro-invasive properties (22, 81–83). This abnormal secretion is in part due to a stress response that results from increased reactive oxygen species downstream of CA (22). Therefore, the presence of amplified centrosomes can also influence the growth of adjacent cells with normal centrosome number in a non-cell-autonomous manner, via secretion of cytokines, growth factors, and extracellular vesicles, suggesting a broader role for cells with CA. Importantly, numerous studies of ADPKD have demonstrated that cystic epithelial cells similarly display secretory phenotypes, resulting in elevated levels of cytokines and other growth factors. These promote a heightened and sustained immune response, which contributes to cyst growth and interstitial fibrosis (84, 85). Thus, we hypothesized that elimination of cells with CA would help attenuate some pathways associated with such secreted factors.

**Figure 7:**
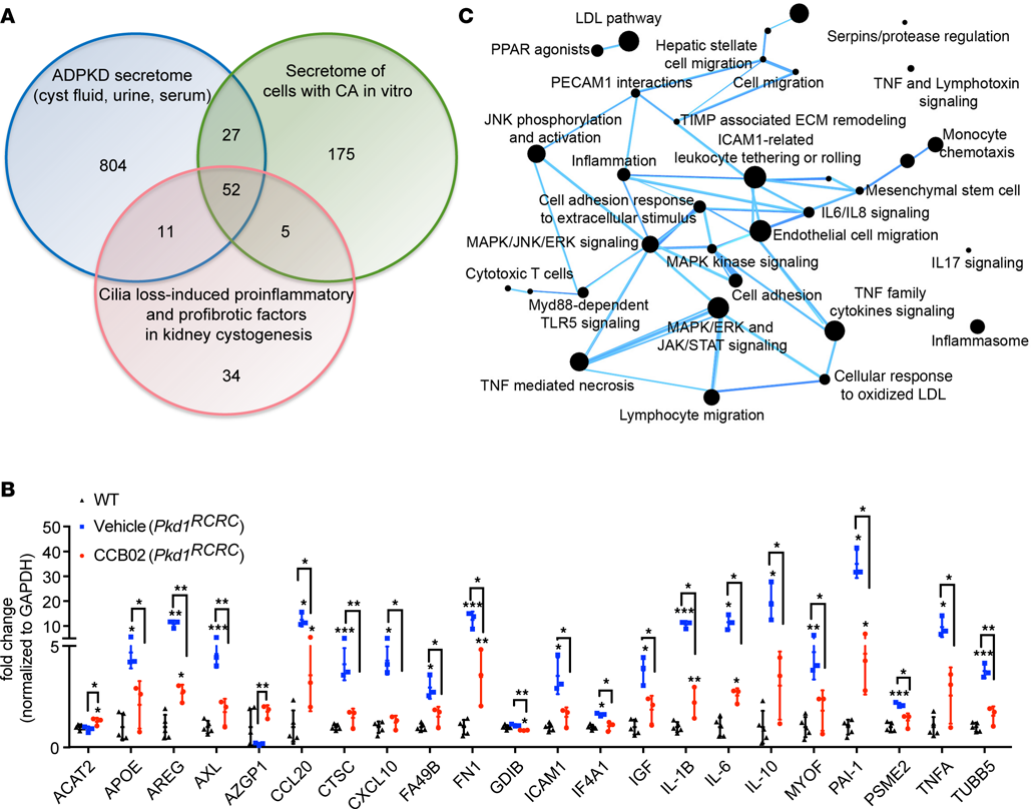
Centrosome clustering inhibitor attenuates pro-inflammatory signaling pathways. (**A**) Venn diagram showing the comparison of secreted factors identified in patients with ADPKD and mouse models, secretome of cells with amplified centrosome, and cilia loss–induced pro-inflammatory and pro-fibrotic factors implicated in cystogenesis. (**B**) Quantification of the relative change in gene expression levels of selected factors in kidneys of wild-type or *Pkd1^RC/RC^* mice treated with CCB02. *n* = 3 mice per group. *P < 0.05, **P < 0.01, ***P < 0.001 (2-tailed). The network of significantly enriched biological themes defined by a CompBio pathway analysis tool, comparing the differentially expressed genes between control and CCB02-treated groups. The size of a sphere is proportional to the CompBio enrichment score of its theme.

To identify the CA-associated factors that may be implicated in driving cyst formation in ADPKD, we compared the secreted factors identified in patients with ADPKD and animal models (85–98) with those characterized by cells with CA in vitro (22, 81) (Figure 7A and Supplemental Data 1). We also included in the analysis proteins that are secreted upon defects in cilia formation in the kidney, as those have also been shown to promote an elevated immune response that exacerbates the cystic disease phenotype and renal fibrosis (85, 99-102). The in silico analysis yielded a total of 52 overlapping proteins (Figure 7A). We then performed quantitative reverse transcription PCR (qRT-PCR) analysis of those genes on kidneys isolated from wild-type and *Pkd1^RC/RC^* mice treated with either vehicle or CCB02 (Figure 7B and Supplemental Figure 8I). As predicted, most of the genes (39/52, 75%) were upregulated in *Pkd1^RC/RC^* mice compared with wild-type control (Figure 7B and Supplemental Figure 8I). Intriguingly, 21/52 (40.4%) showed reduced levels following treatment with CCB02 (Figure 7B and Supplemental Figure 8I). Utilizing the CompBio platform (103, 104), we analyzed the network of these significantly enriched differentially expressed genes to determine the biological themes impacted between the control group and CCB02 treatment group (Figure 7C). The majority of these factors (e.g., TNF-α, IL-1β, IL-6, AREG, PAI1) are broadly involved in extracellular matrix remodeling upon renal injury by driving pro-fibrotic and pro-inflammatory signaling. Some factors (e.g., ICAM1, CCL20, PSME2, AXL) can also enhance proliferative signaling pathways (via MAPK/ERK, JNK, and JAK/STAT signaling, among others) that lead to cyst growth stimulation. Moreover, other elevated factors in our data set are known to regulate immune cell activity and infiltration (e.g., GDIB, CTSC), which are also known to exacerbate and promote cyst growth (102, 105–108). In sum, inhibiting centrosome clustering causes elimination of cells with CA via p53-mediated apoptosis, which attenuates a subset of pro-fibrotic, proliferative, and cystogenic signaling.

## Discussion

In this study, we tested whether centrosome clustering inhibitors can selectively target cells with amplified centrosomes in ADPKD models and attenuate the disease phenotypes. CA has been widely observed in human ADPKD specimens (9, 10), and we determined that this phenomenon is evident in almost half of the cysts. Importantly, analysis of spindle morphology showed that, as these cells divide, they cluster their excess centrosomes and form a pseudo-bipolar mitotic spindle (Figure 1). This robust centrosome clustering mechanism likely helps the cells avoid mitotic catastrophe, allowing them to proliferate and form cysts. The strong centrosome clustering effect was also found in human ADPKD cultured cells, as well as the *Pkd1^RC/RC^* mouse model (Figure 4), which indicates that the survival mechanism of centrosome clustering is conserved in ADPKD, similar to what has been noted for cancer cells with CA (23).

We previously demonstrated that inducing CA in a developing wild-type mouse is sufficient to cause cyst formation, fibrosis, and a decline in renal filtration function (12). Here, we emphasize this point by conditionally driving CA in the kidneys of the slow-onset *Pkd1^RC/RC^* mouse model, at stages when cystogenesis is not yet evident. Indeed, the induction of CA caused accelerated cyst formation and growth, increased interstitial fibrosis, and decreased kidney function (Figure 2). These results highlight the additive effects of CA in the context of ADPKD-inducing mutations. As mutations in ADPKD genes have been shown to disrupt centrosome biogenesis and result in cells with amplified centrosomes (9, 10), the formation of these ectopic centrosomes may therefore act in a feedback loop of sorts, further impacting kidney cystogenesis. So how does a subpopulation of cells with CA have a such a big impact with regard to cytogenesis? Although there are no direct studies of this in ADPKD, it is well established from studies in cancer that a subpopulation of cells with CA in the tumor can play an outsized role in tumorigenesis and metastasis. This occurs due to 2 separate reasons: cell-intrinsic and cell-extrinsic factors. From a cell-intrinsic point of view, the subpopulation of cells with CA divide and form daughter cells that contain defects in genome stability, cell differentiation, invasive behavior, and other processes that promote cell proliferation and tumor growth/metastasis (8, 109). Therefore, that small subpopulation continues to propagate abnormal daughter cells in the population indefinitely. The second way that the small subpopulation of cells with CA plays an outside role is via cell-extrinsic mechanisms. Once again, this information is derived from the cancer field, where it has been nicely shown that CA results in increased secretion of cytokines and pro-inflammatory paracrine signaling factors (22, 81). This means that the small subpopulation of cells with CA secrete factors that promote the proliferation of neighboring cells, even if the neighboring cells have normal centrosome number. Therefore, we predict that cystic epithelial cells with excess centrosomes likely behave similarly with regard to both processes.

The balance of cells with CA in the population is maintained dynamically. A recent study by Dias Louro and colleagues (110) employed a model selection approach to infer patterns of CA and selection in a diverse panel of human cancer cell lines. Their results indicate that the fraction of cells with CA in these populations is at equilibrium, and maintained by a balance of centrosome overproduction and negative selection (apoptosis) of cells with CA, reminiscent of mutation-selection balance. This overproduction-negative selection balance is sufficient to explain the observed differences in the percentage of cell populations with CA. Therefore, we predict that the subpopulation of cells with CA in the cystic epithelia of human and mouse ADPKD (e.g., Figures 1, 4, and 6) is likely maintained by the same overproduction-negative selection balance. Blocking centrosome clustering with the inhibitors may shift this balance, by pushing the pathway more toward negative selection (i.e., apoptosis). Over a long treatment time, this cell death would add up to cause loss of the population of cells with CA. This establishes the hypothesis that eliminating the cells with CA may have beneficial long-term outcomes.

Along these lines, we began by testing the effects of centrosome clustering inhibitors on cultured human ADPKD samples in vitro. We purposefully used different inhibitors — that target different proteins — to avoid any potential issues of off-target effects from any one drug. Our reasoning is that the effects we observe in ADPKD cells should be centrosome clustering dependent, and if different drugs — targeting different pathways of centrosome clustering — result in the same cellular outcomes, that highlights the rigor of our data. Indeed, *PKD1*-null cells with CA treated with either CCB02 or PJ34 formed multipolar and disorganized spindles in mitosis (Figure 3). The ectopic centrosomes were present at most excess spindle poles, and there was a concurrent decrease in the proportion of cells with bipolar spindles, indicating the treatment was effective. The centrosome declustering caused a mitotic delay, activation of the SAC, and elimination of cells with CA from the population via apoptosis. Therefore, centrosome clustering inhibitors are effective in ADPKD cells and can arrest the proliferation of the cystic kidney cells with CA, consistent with what the inhibitors were shown to do in cancer cells and solid tumors (62–64).

Next, we tested the effects of these inhibitors using the slow-onset *Pkd1^RC/RC^* mouse model. Two separate treatment schemes were used in these mice: one starting at 6 months of age, the other at 9 months. This allowed us to examine the functional and morphological consequences of starting the treatment at different stages and severity of cystogenesis. Treatment with these inhibitors blocked the clustering of centrosomes in the cystic kidneys of *Pkd1^RC/RC^* mice, indicating that they are active on kidney cells in vivo. In both cases, treatment of mice with either CCB02 or PJ34 resulted in a significant decrease in kidney size, cyst index, cyst number, and fibrosis, while kidney filtration function was maintained (Figures 4 and 5). These data indicate that inhibiting centrosome clustering during both the slow, progressive growth stage and the rapid cyst expansion stage results in reduced cyst growth, improved kidney morphology, and preserved renal function. These compounds were well tolerated by the animals, who showed better weight management, feeding behavior, and urination than those treated with tolvaptan (Figure 5). This is not surprising since tolvaptan has been reported to cause changes in water retention, fluid secretion, urination, and weight maintenance (74, 75). Importantly, treatment with the centrosome clustering inhibitors, followed by a washout period where no drug was administered for 2 months, showed that the observed improvement in kidney morphology and function were maintained well after the cells with CA were eliminated. Unfortunately, there are no currently available genetic mouse models whereby we can conditionally block centrosome clustering in vivo. Thus, new tools will need to be developed to address the genetic epistasis effects of CA and inhibition of centrosome clustering in future studies.

Finally, we sought to determine whether loss of cells with amplified centrosomes would attenuate their paracrine mediated signaling effects. Several studies have shown that CA promotes the secretion of signaling factors with proliferative, pro-inflammatory, and pro-invasive properties (22, 81–83). The presence of amplified centrosomes in a cell can influence the growth of adjacent cells with normal centrosome number in a non-cell-autonomous manner, via secretion of cytokines, growth factors, and extracellular vesicles. Moreover, numerous studies of ADPKD have shown that cystic epithelial cells display secretory phenotypes, resulting in elevated levels of cytokines and other growth factors. Indeed, recent studies have noted that a heightened and sustained immune response plays a key role in cyst growth and interstitial fibrosis (84, 85). In silico analysis of secreted factors in ADPKD and cancer cells with CA identified 52 common secreted factors, some of which we hypothesized may be impacted by elimination of cells with CA. qRT-PCR analysis indicated that expression of nearly half of those genes is reduced in *Pkd1^RC/RC^* mice treated with a centrosome clustering inhibitor (Figure 7). The majority of these factors have been previously shown to be involved in extracellular matrix remodeling upon renal injury, by modulating pro-fibrotic and pro-inflammatory signaling (85, 99–102). Some secreted factors are known to enhance proliferative signaling pathways that lead to cyst growth stimulation. Moreover, other elevated factors in our dataset are known to regulate immune cell activity and infiltration, which is known to exacerbate and promote cyst growth (102, 105–108). Thus, our qRT-PCR analyses helped shed light on a subset of the CA-dependent paracrine signaling pathways that are active in ADPKD.

In sum, our results indicate that inhibiting centrosome clustering in slow-onset ADPKD mice can block the proliferation of cells with excess centrosomes, which results in reduced cyst growth, improved kidney morphology, and preserved renal function. These data pave the way for testing of such compounds in patients with ADPKD.

## Methods

### Drug administration.

The Pkd1p.R3269C knockin mice (equivalent to human Pkd1p.R3277C mutation, named *Pkd1^RC/RC^* hereafter) were originally generated in C57BL6/J background and were maintained in a heterozygous state (RC/^+^) by mating with C57BL6/J WT mice (The Jackson Laboratory). Genotyping was performed by extracting tail DNA followed by PCR using the following primers (all 3 primer pairs share the same forward primer): Forward: 5′-GTCTGGGTGATAACTGGTGT-3′; PKD1 full length (710 bp): Reverse: 5′-GGACAGCCAAATAGACAGG-3′; PKD1 WT (480 bp): Reverse: 5′-AGGTAACCCTCTGGACTCT-3′; PKD1 mutant (480 bp): Reverse: 5′- AGGTAACCCTCTGGACGCA-3′.

To test the effect of CCB02 and PJ34 on cyst progression at rapid stages of cystogenesis, 9-month-old *Pkd1^RC/RC^* homozygous mice were administered CCB02 (25 mg/kg, dissolved in 10% PEG, 10% citric acid with a final DMSO concentration of 1.5% [v/v]), PJ34 (10 mg/kg, dissolved in PBS), or solvent only (control) via gavage every other day for 2 months. Kidneys from 5 female mice and 5 male mice per group were harvested and analyzed at 11 months of age. To test the effect of CCB02 on cyst progression at rapid stages of cystogenesis, 2-month-old *Pkd1^RC/RC^ mChPlk4* mice were administered with tamoxifen (3 mg, 5 times every other day); 1 month later, *Pkd1^RC/RC^ mChPlk4* mice were administered either CCB02 (25 mg/kg) or solvent only (control) via gavage every other day for 1.5 months. Kidneys from 5 mice per group were harvested and analyzed at 4.5 months of age. To test the effects of CCB02 and tolvaptan on cyst progression at early stages of cystogenesis, 6-month-old *Pkd1^RC/RC^* homozygous mice were divided into 3 groups (control, tolvaptan, CCB02). The mice were administered CCB02 (25 mg/kg), tolvaptan (10 mg/kg, dissolved in PBS), or solvent only (control) every other day via gavage for 5 months. Kidneys from 5 mice per group were harvested and analyzed at 11 months of age. To test the long-term benefits of treatment with the centrosome clustering inhibitors, 9-month-old *Pkd1^RC/RC^* mice were administered CCB02 (25 mg/kg) every other day for 60 days, the treatment was stopped, the mice were followed for an additional 3 months until they reached 14 months of age, and their kidneys (*n* = 4) were harvested and analyzed.

### Evaluation of the number of centrosomes and mitotic spindle morphology.

HK-2 and WT9-12 cells (ATCC) were grown on glass coverslips in DMEM F-12 medium supplemented with 10% FBS. After treatment with CCB02 (2 μM), PJ34 (20 μM), or AZ82 (3 μM), cells were washed with PBS and fixed with ice-cold methanol at –20°C for 5 minutes. Fixed cells were blocked with 0.5% fish gelatin in PBS (block and wash buffer) for 60 minutes at room temperature or at 4°C overnight, then incubated with primary antibodies (centrosomal and SAC markers, as indicated in the figure legends) for 60 minutes at room temperature or 4°C overnight. After incubation, cells were washed with buffer 3 times for 5 minutes and incubated with species-specific Alexa Fluor–conjugated secondary antibodies (Thermo Fisher Scientific) at 1:1,000 dilution for 60 minutes at room temperature. DAPI (1:1,000; Invitrogen) was used to stain DNA. The following primary antibodies were used: rabbit anti-Bub1 (1:50, GeneTex GTX107497), mouse anti–γ-tubulin (1:500, MilliporeSigma T6557), rat anti–α-tubulin (111), mouse anti-CPAP (111), and rabbit anti-Cep152 (111). Images were collected with Leica SP8 laser-scanning confocal microscope. Images were processed using Fiji/ImageJ (NIH), Adobe Photoshop, and Adobe Illustrator.

For quantification of centrosome number in human ADPKD primary cells, 3 ADPKD primary cyst cells from different patients and 1 NHK (normal human kidney) primary renal epithelial cell line were grown on glass coverslips in 1:1 mix of RenaLife Complete Medium (Basal Medium and Additives pack or Complete Kit; Lifeline Cell Technology, LL-0025) and Advanced MEM (Thermo Fisher Scientific 12492), supplemented with 5% FBS. Cells were stained with antibodies against γ-tubulin, α-tubulin, and Cep120. Normal centrosome number was defined as cells containing 1 or 2 foci of γ-tubulin, and CA was characterized by the presence of more than 2 foci per cell.

For quantification of centrosome number in kidney samples, a minimum of 5 tissue sections — a midsagittal section and sections generated in 20 μm increments in both directions from the midsagittal section — were analyzed. Samples were stained with antibodies against γ-tubulin. Normal centrosome number was defined as cells containing 1 or 2 foci of γ-tubulin, and CA was characterized by the presence of more than 2 foci per cell. For evaluation of mitotic spindle morphology, paraffin-embedded kidney specimens were immunostained for γ-tubulin (centrosomes), α-tubulin (microtubules), and DNA (DAPI). Three-dimensional reconstructions of *Z*-stack images were used for the analysis. To determine the fraction of cells with abnormal spindle morphology, we counted all metaphase cells in each kidney section containing more than 1 centrosome and compared that with mitotic cells containing the normal complement of 2 centrosomes.

For evaluation of mitotic spindle morphology in human specimens, paraffin-embedded kidney sections from 8 patients with ADPKD (10 sections/patient, total of 80 sections) and 4 healthy kidney specimens (10 sections/sample, 40 sections total) were immunostained for centrin (centrioles), α-tubulin (microtubules), pHH3, and DNA (DAPI). Three-dimensional reconstructions of *Z*-stack images were used for the analysis. To determine the fraction of cells with abnormal spindle morphology, we counted all metaphase cells in each kidney section containing more than 1 centrosome and compared that with mitotic cells containing the normal complement of 2 centrosomes.

### Sex as a biological variable.

For all mouse experiments, both male and female mice were analyzed. Difference between sexes was not considered as a biological variable.

### Statistics.

Statistical analyses were performed using GraphPad Prism 9.0 or Microsoft Excel. The vertical segments in box plots show the first quartile, median, and third quartile. The whiskers on both ends represent the maximum and minimum values for each data set analyzed. Collected data were examined by 1- or 2-way ANOVA or 2-tailed unpaired *t* test as specified in the figure legends. Data distribution was assumed to be normal, but this was not formally tested. Statistical significance was set as *P* < 0.05.

### Study approval.

All animal studies were performed following guidelines of the Institutional Animal Care and Use Committee at Washington University and the NIH and with the approval of the Institutional Animal Care and Use Committee at Washington University.

### Data availability.

Values for all data points found in graphs are in the Supporting Data Values file.

Further information can be found in Supplemental Methods.

## Author contributions

TC and MRM conceived and designed the study. TC performed the vast majority of experiments presented in this study. AM and JG conceived of, and performed, the cell culture experiments. EL performed the qRT-PCR experiments and data analysis. KS assisted with mouse crosses and genotyping. TC and MRM wrote and edited the manuscript. All authors reviewed the final version of the manuscript.

## Supplementary Material

Supplemental data

Supplemental data set 2

Supplemental data sets and 1

Unedited blot and gel images

Supporting data values

## Version 1 01/23/2024

In-Press Preview

## Version 2 02/22/2024

Electronic publication

## Version 3 08/23/2024

Fixed mouse strain to C57BL6/J in Methods
