# Supplementary material for: Inhibiting centrosome clustering reduces cystogenesis and improves kidney function in autosomal dominant polycystic kidney disease
Source: JCI Insight. 2024 Feb 22;9(4):e172047. doi: 10.1172/jci.insight.172047 (PMC10967408; doi:10.1172/jci.insight.172047)
Supplement: Supplemental data [file jciinsight-9-172047-s008.pdf]

Supplemental Methods:

Analysis of human ADPKD specimens:

Human kidney samples were obtained from archived materials stored in the Kidney Translational Research Center (Washington University in St. Louis) according to the guidelines of a protocol approved by the Institutional Review Board of the Washington University School of Medicine. Renal specimens were fixed in formalin and embedded in paraffin, and 10 μm sections were cut and placed onto microscope slides. Sections from eight ADPKD patients, with ages ranging from 56–75 yr, were used for analysis. Specimens were processed and stained with antibodies to mark centrosomes and mitotic spindles as described below.

Histology, Immunohistochemistry, and Immunofluorescence:

Both kidneys were isolated from each adult mouse as previously described (1) and fixed in one of two ways, depending on the downstream application. Specimens were fixed in 4% paraformaldehyde in PBS for 24 h at 4 °C, then embedded in paraffin. Samples were cut into 10 μm sections using a microtome (RM2125 RTS; Leica), placed onto microscope slides (Thermo Fisher Scientific), and stored at room temperature. For spindle morphology analysis, samples were cut into 15 μm sections. Alternatively, freshly isolated kidneys were placed in appropriately sized cryomolds (Tissue-Tek), immersed in Optimal Cutting Temperature compound (Tissue-Tek), and placed in a dry ice/ethanol bath for 10 min. Frozen samples were cut into 10 μm sections using a cryostat (CM1850; Leica), placed onto microscope slides, and stored at −80 °C. Histological staining of

paraffin-embedded sections with hematoxylin and eosin (H&E) was performed using standard protocols.

For immunohistochemistry of paraffin-embedded sections, antigen unmasking was performed by boiling the slides in antigen-retrieval buffer (10 mM Tris Base, 1 mM EDTA, and 0.05% Tween-20, pH 9.0) for 30 min. For immunostaining of cryopreserved sections, samples were fixed using precooled (−20 °C) methanol for 10 min. After fixation, both types of fixed samples were pre-extracted with 0.05% Triton X-100 in PBS for 10 min at room temperature, incubated in blocking buffer (3.0% BSA and 0.05% Triton X-100 in PBS) for 1 h, followed by staining with primary and secondary antibodies. The complete list of antibodies used in this study is provided in Supplemental Table 1. All Alexa Fluor dye–conjugated secondary antibodies were obtained from Thermo Fisher Scientific and utilized at a final dilution of 1:500. Nuclei were stained with DAPI, and specimens mounted onto slides using Mowiol antifade mounting medium containing n-propyl gallate (Sigma-Aldrich). Images were captured using a Nikon Eclipse Ti-E inverted confocal microscope equipped with 10× Plan Fluor (0.30 NA), 20× Plan Apo air (0.75 NA), 60× Plan Fluor oil immersion (1.4 NA), or 100× Plan Fluor oil immersion (1.45 NA) objectives (Nikon). A series of digital optical sections (z-stacks) were captured using Hamamatsu ORCA-Fusion Digital CMOS camera at room temperature, and three-dimensional image reconstructions were produced. Images were processed and analyzed using Elements AR 5.21 (Nikon) and Photoshop software (Adobe).

Evaluation of Kidney Fibrosis:

Masson's trichrome stain and immunofluorescence staining using α-SMA were used for the assessment of fibrosis. Calculation of the total kidney area and the percentage area covered by α-SMA staining was utilized to measure the area covered by fibrotic cells. Multiple fields from each kidney section were imaged using a 20× objective. 15 randomly selected fields from three tissue sections per sample were quantified. The data are expressed as the mean area of α-SMA-positive foci per unit area (squared millimeter).

Quantification of cyst number and cyst index:

Sagittal kidney sections were stained with hematoxylin and eosin (H&E) and examined by light microscopy. ImageJ software was used to measure the area of each cyst and to quantify the total numbers of cysts. A dilated tubule was counted as a cyst if its area exceeded 0.02% of the total kidney area. Cysts were scored in three sections from each kidney sample.

BUN and serum creatinine measurements:

Blood serum was obtained by centrifugation (6,000 x g, 15 minutes at 4 °C) of blood samples isolated via sub-mandibular bleeding and used for the BUN assay (BUN-Urea, BioAssay Systems) following the manufacturer’s protocol. Creatinine levels were quantified by HPLC (UAB-UCSD O'Brien Center, The University of Alabama at Birmingham).

Analysis of DNA damage and apoptosis:

Antibodies against γ-H2AX were used to identify DNA damage, and p53 staining was performed as described above. Images were captured using a Nikon Eclipse Ti-E inverted confocal microscope equipped with 40× Plan Fluor oil immersion (1.4 NA). The percentage of γ-H2AX-positive cells or nuclear p53-positive cells in each kidney section were determined. Briefly, ImageJ software was used to count the number of total cells (based on the DAPI blue stain) and the number of γ-H2AX-positive cells or nuclear p53-positive cells (based on the green stain) in each field. The number of green cells was then divided by the number of total cells for each field.

The TUNEL method was used to quantify apoptosis following the manufacturer's instructions (In Situ Cell Death Detection kit; Roche). The percentage of TUNEL-positive cells in each kidney was quantified as described above. All calculations were done from a minimum of five sections from experimental and control animals.

RNA isolation, RT-PCR and quantitative PCR:

Total RNA was isolated from mouse kidneys using Direct-zol™ RNA MiniPrep Plus (Zymo Research). 2 µg of RNA was reverse-transcribed using a High Capacity cDNA Reverse Transcription Kit (Applied Biosystems, ThermoFisher Scientific) according to the manufacturer protocol. Real-time PCR was performed with SYBR Select Master Mix (Applied Biosystems) in a 96- or 384-well plate format. 50 ng of cDNA was used in 10 ul final volume and the reactions run at the standard cycling mode recommended by the manufacturer, using QuantStudio 6 Flex system (Applied Biosystems). GAPDH was used

as an endogenous control and data analyzed with ΔΔCt method. The complete list of primers used in this study is provided in Supplemental Data 2.

Immunoblot analysis

HK-2 and WT9-12 cells were grown in 6-well plates in DMEM/F-12 medium supplemented with 10% FBS. Cells were treated with DMSO or CCB02 (2 μM) for 24 h, washed with PBS and lysed in 1x SDS sample buffer. Kidneys were collected and homogenized in T-PER lysis buffer (Thermo Fisher Scientific) supplemented with 2X protease cocktail (Pierce) and 3X phosphatase inhibitors (Roche). The homogenized lysate was incubated at 4°C for 20 min and cell debris cleared by centrifugation at 12,000 g for 10 min. 50 μg of protein lysate from each group was analyzed by SDS-PAGE. Membranes were incubated overnight at 4°C with the primary antibodies (Supplemental Table 1). After incubation, membranes were washed with 1 x TBS plus 0.05% Tween-20 buffer three times for 10 min and incubated with horseradish peroxidase-conjugated secondary antibodies (Jackson ImmunoResearch Laboratories) at 1:1000 dilution for 60 min at room temperature. Membranes were imaged on a BioRad scanner.

CompBio analyses:

The secreted factors identified by PCR were further analyzed using the CompBio (COmprehensive Multi-omics Platform for Biological InterpretatiOn) software package. CompBio analyzes single or multi-omic data entities (genes, proteins, microbes, metabolites, miRNA) and deliver a holistic, contextual map of the core biological concepts and themes associated with those entities. Conditional probability analysis calculated the

statistical enrichment of biological concepts (processes/pathways) over those that occur by random sampling. Related concepts built from the list of differentially expressed entities were further clustered into higher-level themes (e.g., biological pathways/processes, cell types and structures, etc.).

References:

1. Dionne LK, Shim K, Hoshi M, Cheng T, Wang J, Marthiens V, et al. Centrosome amplification disrupts renal development and causes cystogenesis. *J Cell Biol.* 2018;217(7):2485-501.

Supplemental Figure 1

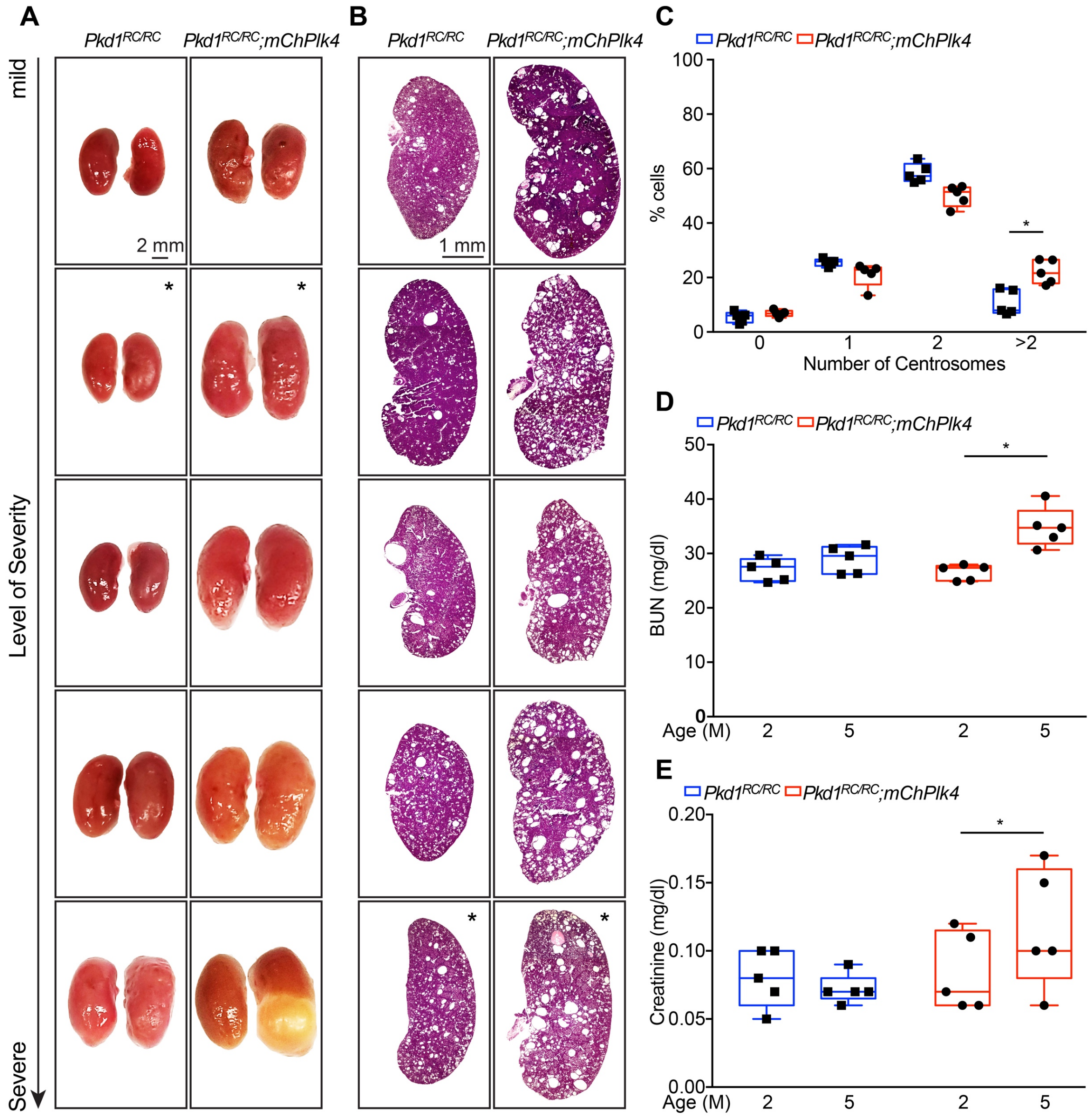

Supplemental Figure 1. **(A** and **B)** Images of whole kidneys and H&E-stained sections of all *Pkd1*$^{RC/RC}$ and *Pkd1*$^{RC/RC}$*;mChPlk4* mice at 5 months of age that were analyzed in this study, organized by level of disease severity. Images containing an asterisk were the examples utilized in the main figure. (**C)** Quantification of centrosome number in cyst-lining cells of *Pkd1*$^{RC/RC}$ and *Pkd1*$^{RC/RC}$*;mChPlk4* kidneys. (**D)** Analysis of absolute levels of blood urea nitrogen (BUN) and (**E)** serum creatinine levels of *Pkd1*$^{RC/RC}$ and *Pkd1*$^{RC/RC}$*;mChPlk4* mice at 5 months. N = 5 mice per group for all experiments. * = $p<0.05$ (one-way ANOVA).

Supplemental Figure 2

A

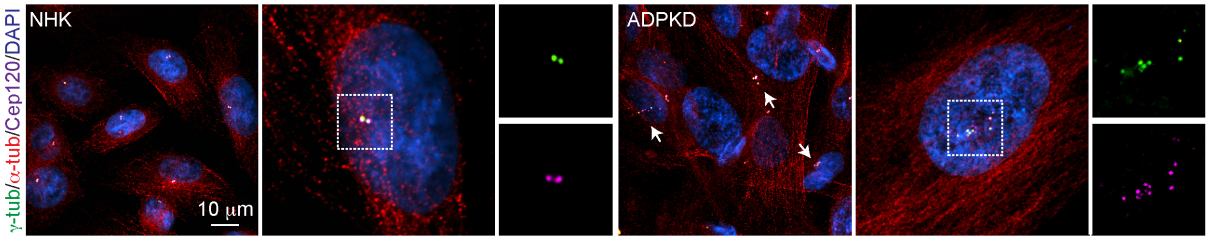

B

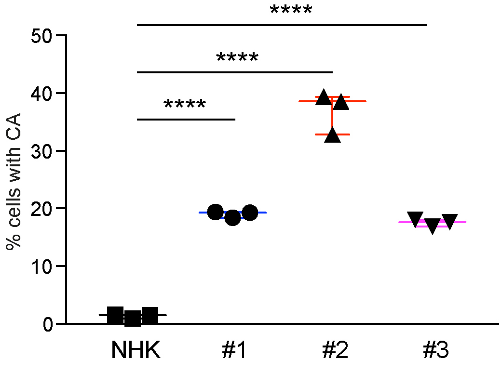

Supplemental Figure 2. (A) Representative immunofluorescence images of primary normal human kidney (NHK) and ADPKD cells stained with antibodies that mark centrioles (Cep120), centrosomes (γ-tubulin), microtubules (α-tubulin) and DNA (DAPI). White arrows point to cells with amplified centrosomes. **(B)** Quantification of the percentage of cells with amplified centrosomes in three separate ADPKD cells. N = 428 cells (NHK), 332 (ADPKD #1), 357 (ADPKD #2) and 330 (ADPKD #3). Data are from three independent experiments. **** = $p<0.0001$ (two-way ANOVA).

Supplemental Figure 3

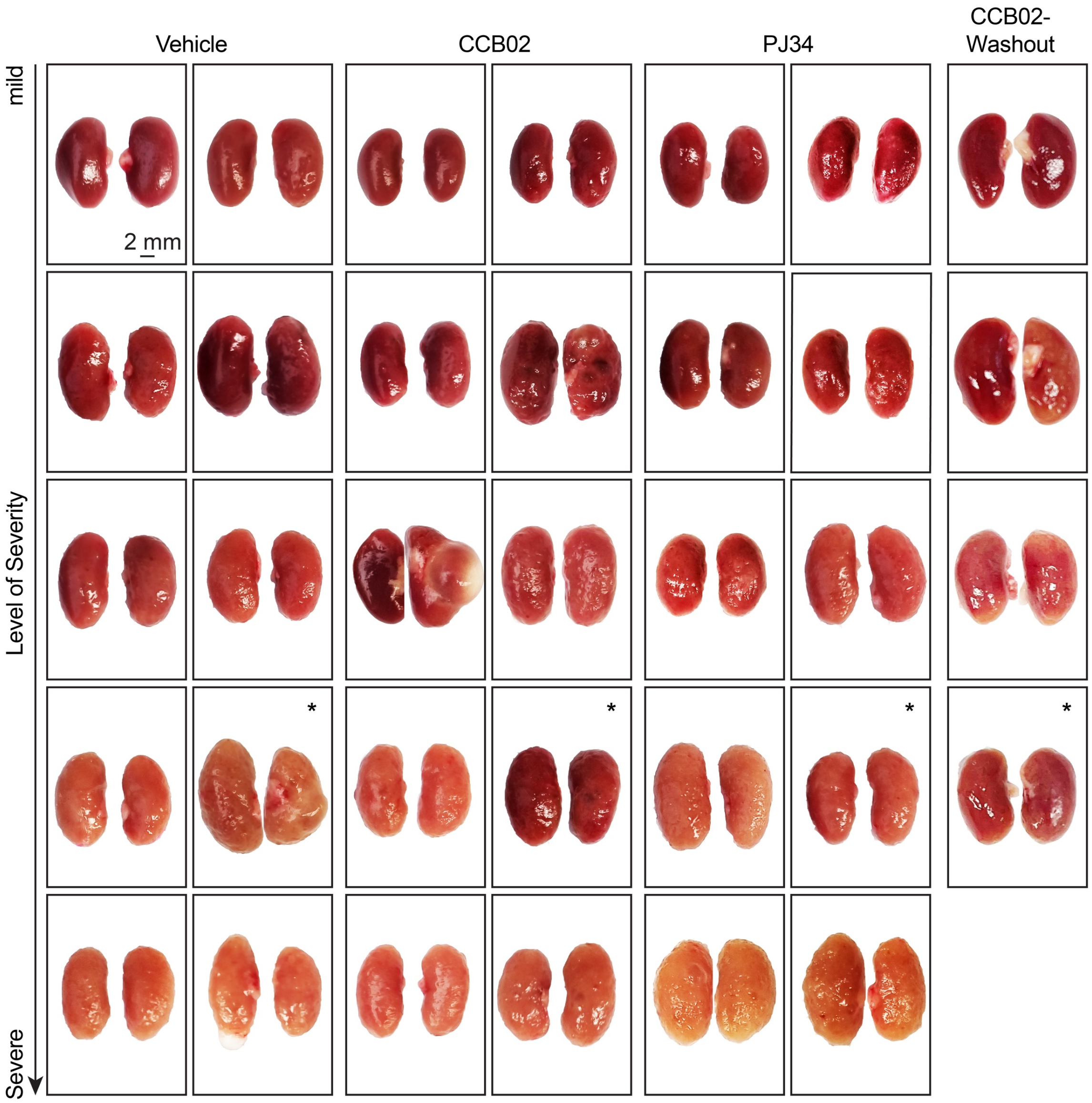

Supplemental Figure 3. Images of whole kidneys from all *Pkd1*$^{RC/RC}$ mice treated with either CCB02, PJ34, and post-washout that were analyzed in this study organized by level of disease severity. Images containing an asterisk were the examples utilized in the main figure.

Supplemental Figure 4

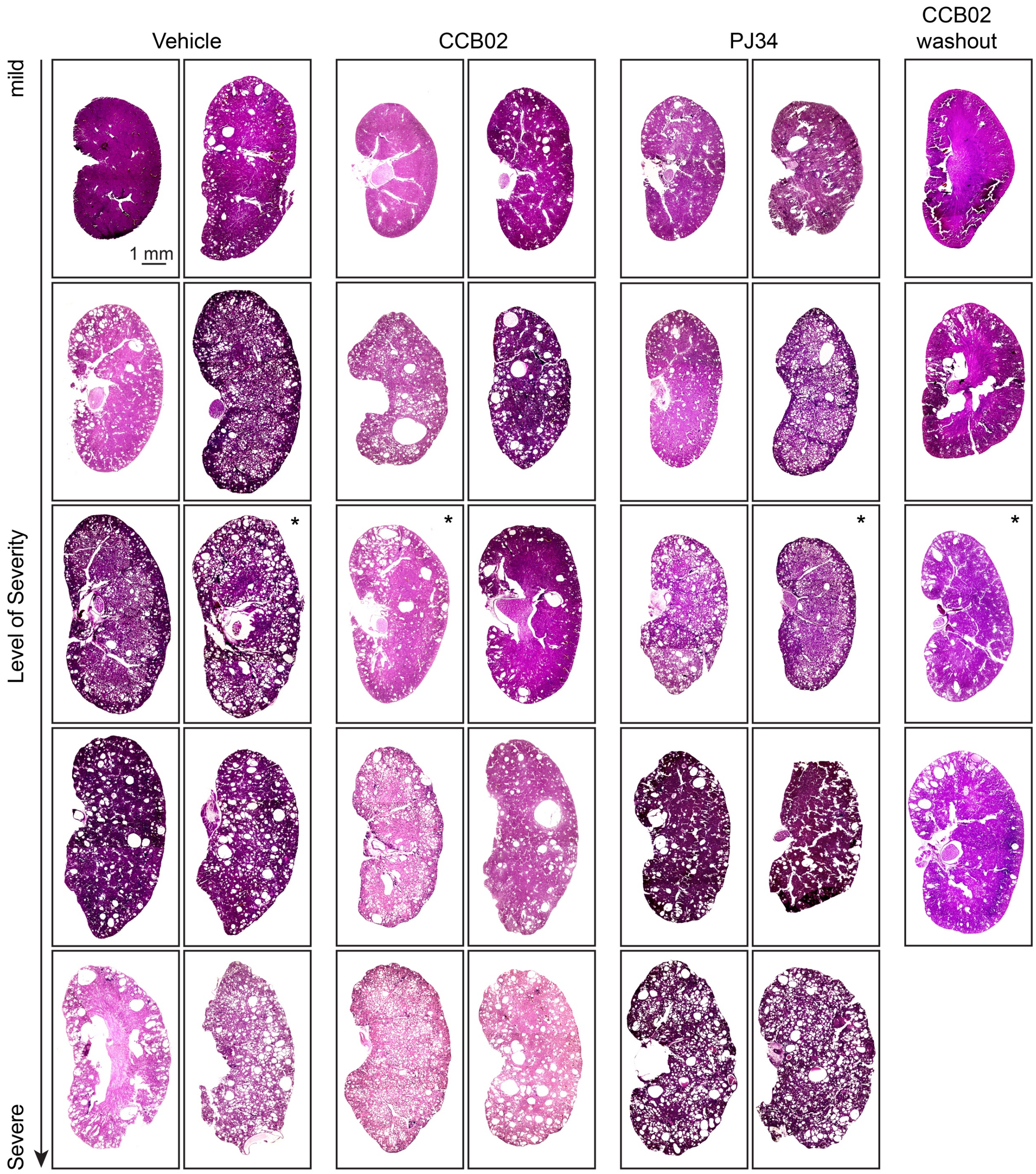

Supplemental Figure 4. H&E-stained images of kidney sections from all *Pkd1*$^{RC/RC}$ mice treated with either CCB02, PJ34, and post-washout that were analyzed in this study organized by level of disease severity. Images containing an asterisk were the examples utilized in the main figure.

Supplemental Figure 5

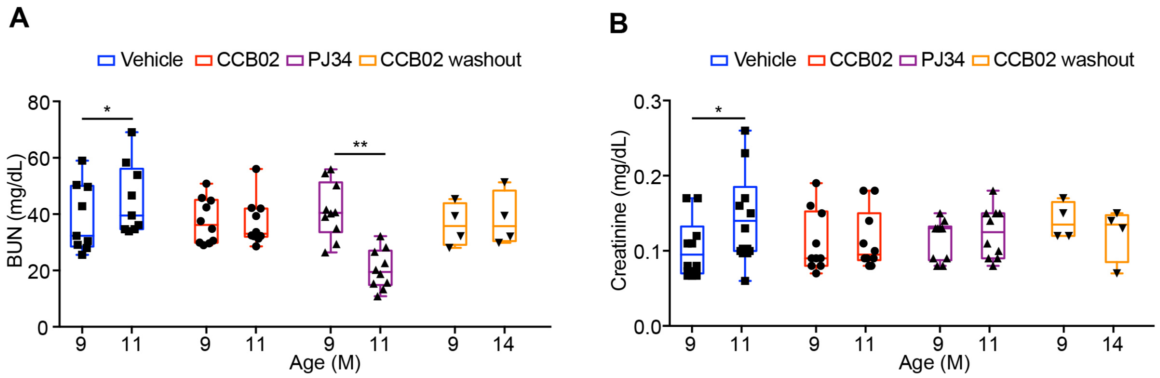

Supplemental Figure 5. (A) Quantification of absolute levels of blood urea nitrogen (BUN) and (**B)** serum creatinine levels of *Pkd1*$^{RC/RC}$ mice treated with CCB02 or PJ34 at 9 months, 11 months, and post-washout (14 months). N = 10 mice each for Vehicle, CCB02 and PJ34; N = 4 animals for CCB02 washout group. * = p<0.05, ** = p<0.01 (one-way ANOVA).

Supplemental Figure 6

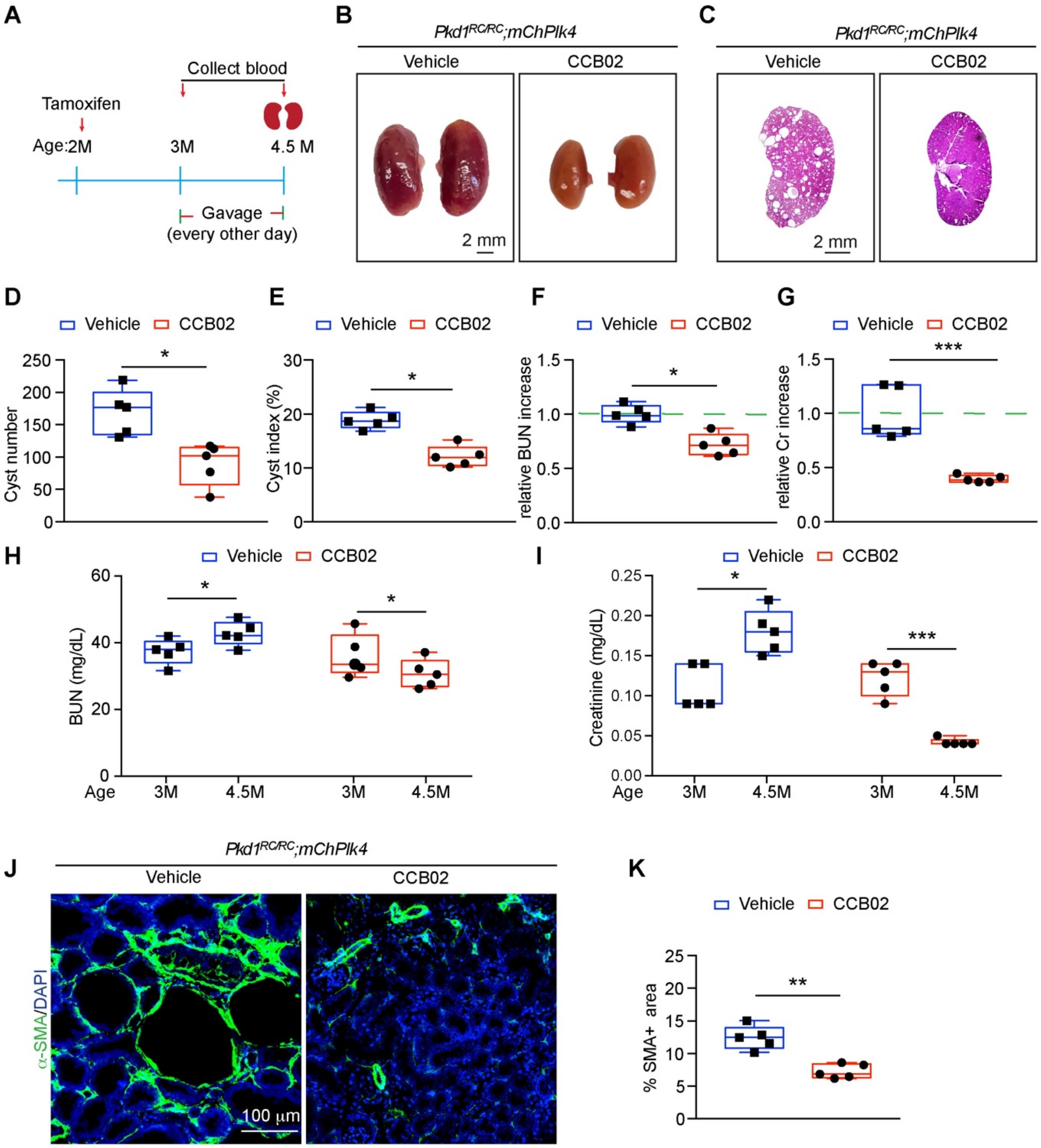

Supplemental Figure 6. (A) Schematic representation of CCB02 treatment scheme in *Pkd1*$^{RC/RC}$;*mChPlk4* mice. **(B** and **C)** Images of whole kidneys and H&E-stained sections of *Pkd1*$^{RC/RC}$;*mChPlk4* mice at 4.5 months of age after treatment with CCB02. **(D)** Quantification of cyst number and **(E)** fractional cyst area per kidney section in treated mice. **(F** and **H)** Analysis of relative blood urea nitrogen (BUN) and **(G** and **I)** serum creatinine levels at 4.5 months. **(J)** Immunofluorescence staining with α-smooth muscle actin (SMA; myofibroblasts) and DNA (DAPI) of kidney sections following the indicated treatment regimen. **(K)** Quantification of the fraction of α-SMA-positive area. N = 5 mice each group. * = $p<0.05$, ** = $p<0.01$, *** = $p<0.001$(one-way ANOVA).

Supplemental Figure 7

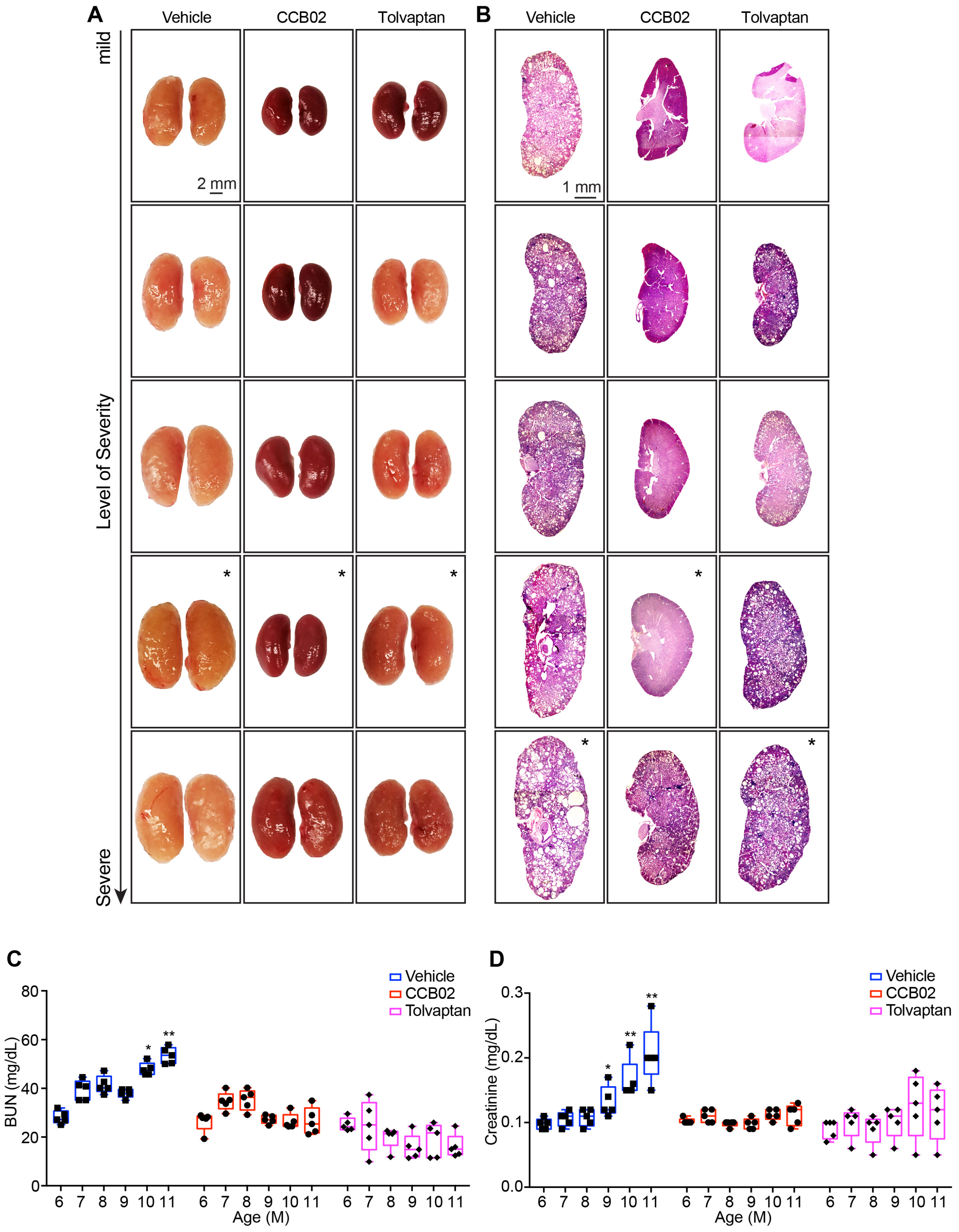

Supplemental Figure 7. (A) Images of whole kidneys and (**B)** H&E-stained sections from all *Pkd1*$^{RC/RC}$ mice treated with CCB02 or Tolvaptan that were analyzed in this study, organized by level of disease severity. Images containing an asterisk were the examples shown in the main figure. (**C** and **D**) Quantification of absolute levels of blood urea nitrogen (BUN) and serum creatinine levels of *Pkd1*$^{RC/RC}$ mice during treatment timeline. N = 5 mice per group for all experiments. * = $p<0.05$, ** = $p<0.01$ (one-way ANOVA).

Supplemental Figure 8

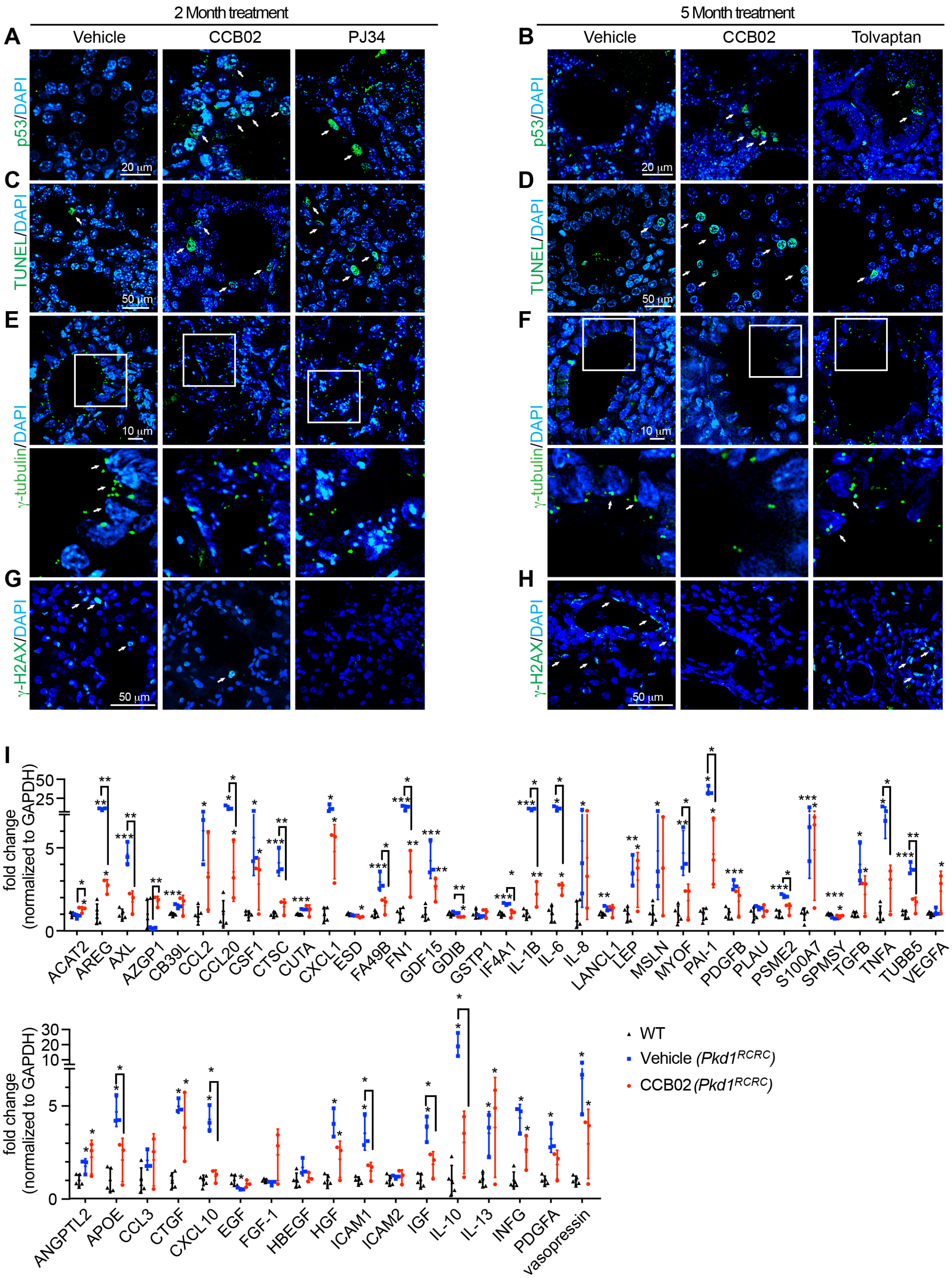

Supplemental Figure 8. Immunofluorescence staining of kidney sections from *Pkd1*$^{RC/RC}$ mice treated for either CCB02 or PJ34 for 2 months, or with CCB02 or Tolvaptan for 5 months. Kidney sections from 11 month-old mice were stained with antibodies to highlight nuclear p53 (**A** and **B**), TUNEL-positive cells (**C** and **D**), centrosomes (**E** and **F**), γ-H2AX (**G** and **H**), and DNA (DAPI). Arrows point to examples of positive cells in each case. White box in panels **E** and **F** highlight magnified regions shown below. (**I)** qPCR-based quantification of the relative change in gene expression levels of all 52 factors identified from *in silico* analysis. N = 3 mice per group. * = $p<0.05$, ** = $p<0.01$, *** = $p<0.001$ (t-test).

Supplemental Figure 9

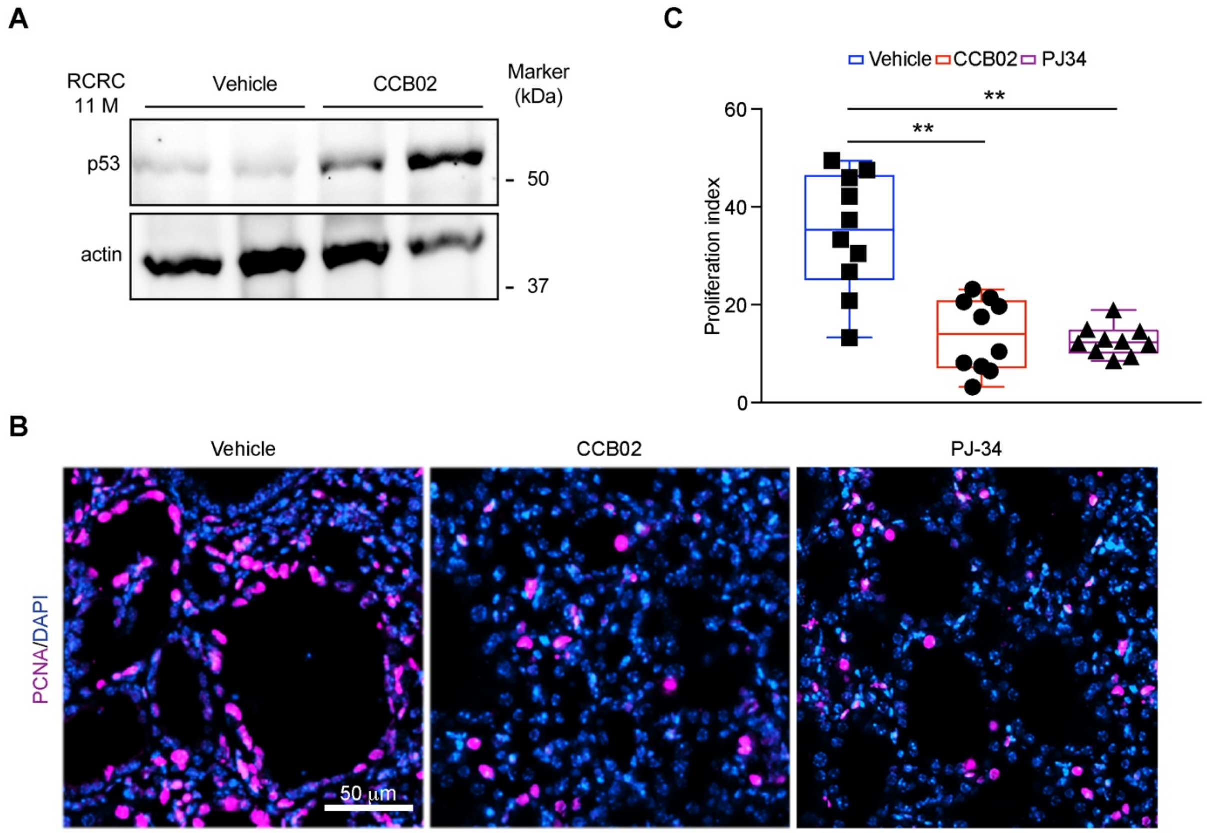

Supplemental Figure 9. (**A**) Immunoblot of p53 of kidney lysate from 11-month-old *Pkd1*$^{RC/RC}$ mice treated for either vehicle or CCB02 for 2 months. (**B**) Immunofluorescence staining of kidney sections from *Pkd1*$^{RC/RC}$ mice treated for either CCB02 or PJ34 for 2 months. Kidney sections from 11 month-old mice were stained with antibodies to PCNA. (**C)** Quantification of the percentage of cells with PCNA staining. N = 10 mice each group. * = p<0.05, ** = p<0.01, *** = p<0.001 (one-way ANOVA).

Supplemental Table 1: List of antibodies used in this study

Antibodies (clone #)	Product details	Dilution
Mouse anti centrin (20H5)	EMD Milipore (04-1624)	1:200
Mouse anti γ-tubulin (GTU-88)	Sigma-Aldrich (T5326)	1:500
Rat anti α-tubulin	Santa Cruz (sc-53029)	1:500
Rabbit anti p53	Leica (NCL-L-p53-CM5p)	1:50
Rabbit anti γ-H2AX (Ser139)	Cell Signaling (9718S)	1:100
Rat anti mCherry (16D7)	Invitrogen (M11217)	1:500
Mouse anti α-SMA	Sigma-Aldrich (A2547)	1:500
Rat anti-Cep120	Produced in-house	1:500
anti-Cep152	REF: 89	1:500
anti-Bub1	#GTX107497	1:50
Mouse anti-BubR1	BD Bioscience (612502)	1:1000
Mouse anti-Mdm2	Santa Cruz (sc-965)	1:1000
Mouse anti-P53	EMD Milipore (OP43)	1:1000
Rabbit anti-p21	ProteinTech (10355-1-AP)	1:1000
Rabbit anti-cleaved caspase3	Cell Signaling (9664)	1:500
Rat anti-caspase2	EMD Milipore (MAB3507)	1:1000
Mouse anti-actin	Sigma-Aldrich (A4700)	1:1000
Rabbit anti phospho-Histone H3	Cell Signaling (9701)	1:200
Mouse anti phospho-Histone H3	Cell Signaling (9706)	1:200

Supplemental Data 1: Full list and comparison of secreted factors identified in various studies, data used in Figure 7 A and B.

Supplemental Data 2: Primer list for qRT-PCR of secreted factors.
