# Supplementary figures and images for: Inhibiting centrosome clustering reduces cystogenesis and improves kidney function in autosomal dominant polycystic kidney disease
Source: JCI Insight. 2024 Feb 22;9(4):e172047. doi: 10.1172/jci.insight.172047 (PMC10967408; doi:10.1172/jci.insight.172047)

WB blots for Figure 3H

BubR1

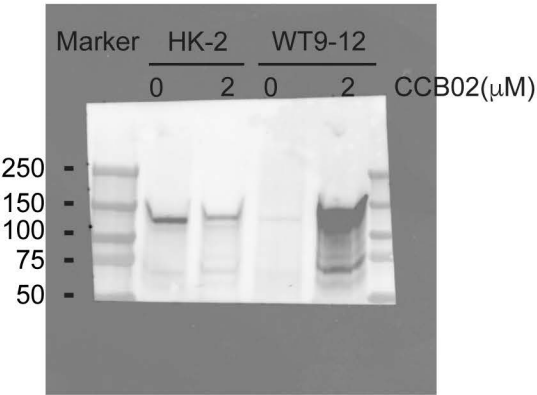

pro-Casp2

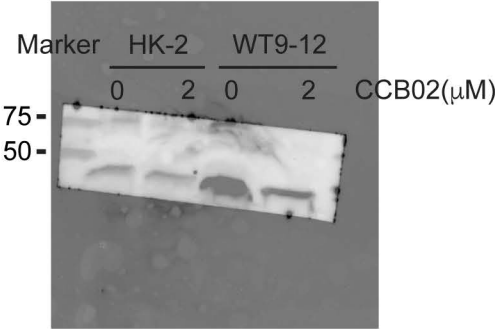

Mdm2

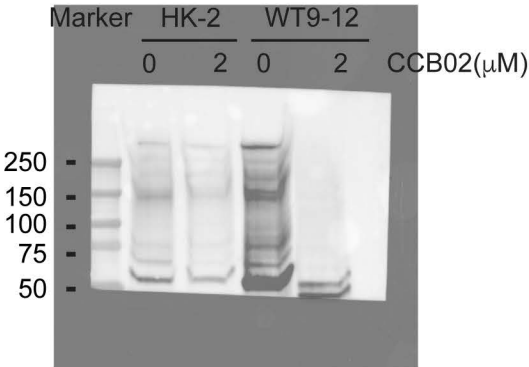

P53

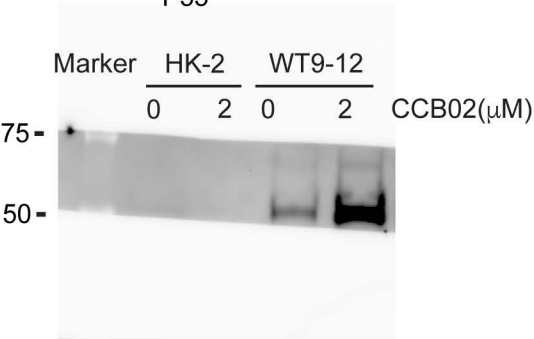

P21

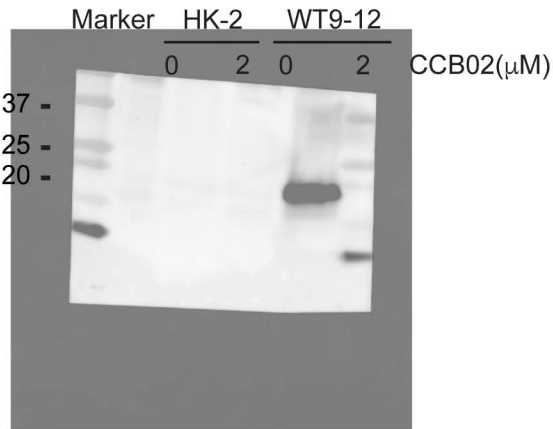

CC3

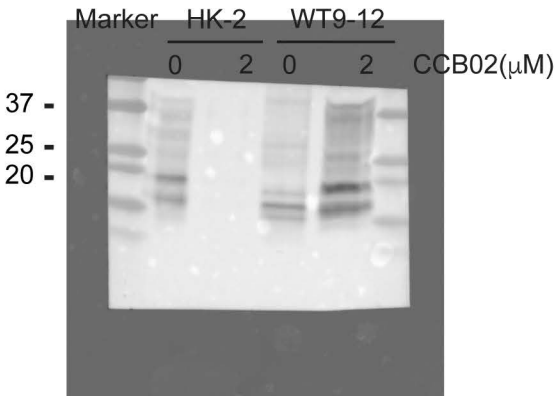

actin

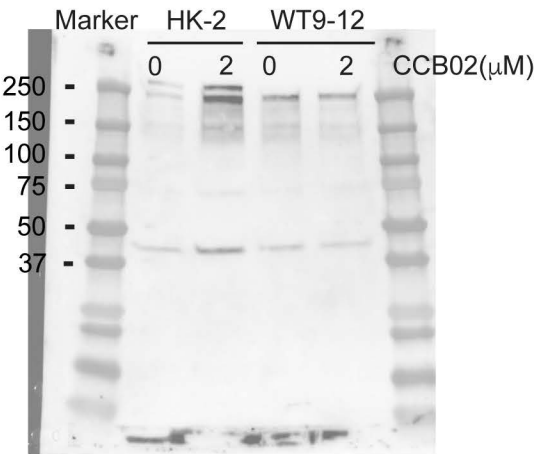

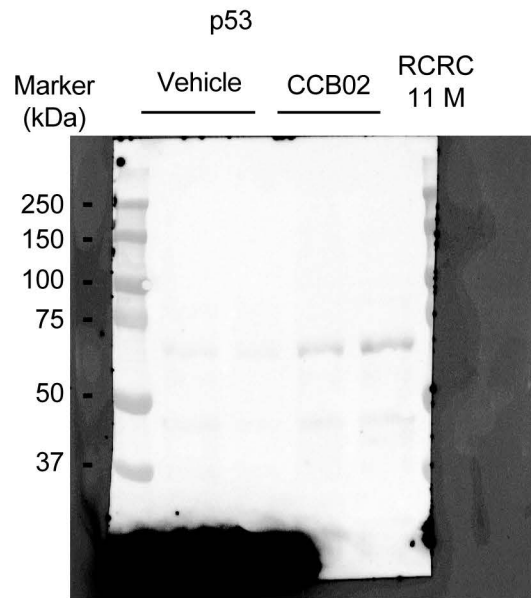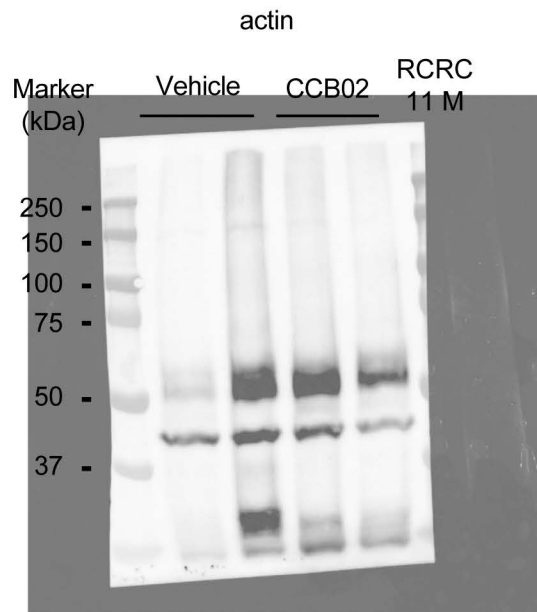

Supplement: Unedited blot and gel images [file jciinsight-9-172047-s011.pdf]
